# Translation initiation of leaderless and polycistronic transcripts in mammalian mitochondria

**DOI:** 10.1093/nar/gkac1233

**Published:** 2023-01-11

**Authors:** Cristina Remes, Anas Khawaja, Sarah F Pearce, Adam M Dinan, Shreekara Gopalakrishna, Miriam Cipullo, Vasileios Kyriakidis, Jingdian Zhang, Xaquin Castro Dopico, Olessya Yukhnovets, Ilian Atanassov, Andrew E Firth, Barry Cooperman, Joanna Rorbach

**Affiliations:** Department of Chemistry, University of Pennsylvania, Philadelphia, PA 19104, USA; Department of Medical Biochemistry and Biophysics, Division of Molecular Metabolism, Karolinska Institutet, Stockholm 17165, Sweden; Max Planck Institute Biology of Ageing - Karolinska Institutet Laboratory, Karolinska Institutet, Stockholm, Sweden; Department of Medical Biochemistry and Biophysics, Division of Molecular Metabolism, Karolinska Institutet, Stockholm 17165, Sweden; Max Planck Institute Biology of Ageing - Karolinska Institutet Laboratory, Karolinska Institutet, Stockholm, Sweden; Department of Pathology, University of Cambridge, Cambridge, UK; Department of Medical Biochemistry and Biophysics, Division of Molecular Metabolism, Karolinska Institutet, Stockholm 17165, Sweden; Max Planck Institute Biology of Ageing - Karolinska Institutet Laboratory, Karolinska Institutet, Stockholm, Sweden; Department of Medical Biochemistry and Biophysics, Division of Molecular Metabolism, Karolinska Institutet, Stockholm 17165, Sweden; Max Planck Institute Biology of Ageing - Karolinska Institutet Laboratory, Karolinska Institutet, Stockholm, Sweden; Department of Medical Biochemistry and Biophysics, Division of Molecular Metabolism, Karolinska Institutet, Stockholm 17165, Sweden; Max Planck Institute Biology of Ageing - Karolinska Institutet Laboratory, Karolinska Institutet, Stockholm, Sweden; Department of Medical Biochemistry and Biophysics, Division of Molecular Metabolism, Karolinska Institutet, Stockholm 17165, Sweden; Max Planck Institute Biology of Ageing - Karolinska Institutet Laboratory, Karolinska Institutet, Stockholm, Sweden; Department of Microbiology, Tumor & Cell Biology, Karolinska Institutet, Stockholm 171 77, Sweden; RWTH Aachen, I. Physikalisches Institut (IA), Aachen, Germany; Forschungszentrum Jülich, Institute of Complex Systems ICS-5, Jülich, Germany; Proteomics Core Facility, Max-Planck-Institute for Biology of Ageing, Joseph-Stelzmann-Str. 9b, 50931 Cologne, Germany; Department of Pathology, University of Cambridge, Cambridge, UK; Department of Chemistry, University of Pennsylvania, Philadelphia, PA 19104, USA; Department of Medical Biochemistry and Biophysics, Division of Molecular Metabolism, Karolinska Institutet, Stockholm 17165, Sweden; Max Planck Institute Biology of Ageing - Karolinska Institutet Laboratory, Karolinska Institutet, Stockholm, Sweden; STIAS: Stellenbosch Institute for Advanced Study at Stellenbosch University, Marais Rd, Stellenbosch 7600, South Africa

## Abstract

The synthesis of mitochondrial OXPHOS complexes is central to cellular metabolism, yet many molecular details of mitochondrial translation remain elusive. It has been commonly held view that translation initiation in human mitochondria proceeded in a manner similar to bacterial systems, with the mitoribosomal small subunit bound to the initiation factors, mtIF2 and mtIF3, along with initiator tRNA and an mRNA. However, unlike in bacteria, most human mitochondrial mRNAs lack 5′ leader sequences that can mediate small subunit binding, raising the question of how leaderless mRNAs are recognized by mitoribosomes. By using novel *in vitro* mitochondrial translation initiation assays, alongside biochemical and genetic characterization of cellular knockouts of mitochondrial translation factors, we describe unique features of translation initiation in human mitochondria. We show that *in vitro*, leaderless mRNA transcripts can be loaded directly onto assembled 55S mitoribosomes, but not onto the mitoribosomal small subunit (28S), in a manner that requires initiator fMet-tRNA^Met^ binding. In addition, we demonstrate that in human cells and *in vitro*, mtIF3 activity is not required for translation of leaderless mitochondrial transcripts but is essential for translation of ATP6 in the case of the bicistronic ATP8/ATP6 transcript. Furthermore, we show that mtIF2 is indispensable for mitochondrial protein synthesis. Our results demonstrate an important evolutionary divergence of the mitochondrial translation system and further our fundamental understanding of a process central to eukaryotic metabolism.

## INTRODUCTION

Translation initiation is a tightly controlled, rate-limiting step of protein synthesis, in which the initiation complex is formed on the start codon of the mRNA. In mitochondria, translation is achieved by specialized mitoribosomes. These macromolecular machines synthesize 13 proteins, all of which are hydrophobic components of the oxidative phosphorylation (OXPHOS) system, essential for cellular energy production. Given cellular and organism metabolic needs, this system impacts metabolic disease pathology (1).

Over evolutionary time, human mitoribosomes have diverged considerably from their bacterial ancestors (2–5), as has the associated translational machinery, which has acquired many unique features. For instance, most bacterial mRNA transcripts carry a Shine-Dalgarno sequence upstream of the start codon that facilitates correct start codon selection by the ribosomal small subunit (SSU) (6). In contrast, the 11 human mitochondrial DNA-encoded transcripts are leaderless, either lacking or carrying very short 5′ UTRs (2).

In prokaryotes, leaderless mRNAs have been reported to bind with a higher affinity for the 70S monosome than to the SSU (7) and interestingly, this interaction can occur in the absence of initiation factors (8,9). While non-canonical translation initiation pathways on monosomes have been relatively well characterized for bacteria (7–10) related mechanisms in mammalian mitochondria remain to be described.

Mitochondrial translation initiation factors are highly divergent from their bacterial counterparts: IF1 is absent in mitochondria and the nuclear-encoded mitochondrial IF2 (mtIF2) and IF3 (mtIF3), both homologous to bacterial initiation factors, have acquired specific extensions and insertions (11,12). In bacteria, the main role of IF2 is selecting the correct initiator tRNA and promoting association of the two ribosomal subunits with the help of its mostly unstructured N-terminal domain (13). Structural and biochemical studies have shown that in mammalian mitochondria, mtIF2 facilitates selection of the formylated initiator fMet-tRNA^fMet^ and replaces the function of bacterial IF1 via a unique 37 aa domain insertion (14,15). Since mtIF2 lacks the corresponding bacterial N terminal domain, it is not clear whether this factor has a subunit association effect, and so far, there is no experimental study addressing this question.

Bacterial IF3 has several specific functions at the interface of ribosome recycling and translation initiation. It binds to the SSU after translation termination to avoid its re-association with the LSU (16), and facilitates accommodation of the initiator tRNA in the P site of the ribosome (17). In addition, IF3 has been proposed to be essential for the 70S-scanning mechanism on the polycistronic transcript, promoting initiation of the downstream cistron (18).

Recent high-resolution structures of the mitochondrial pre-initiation and the complete initiation complexes have elucidated several important details of translation initiation in the organelle (15,19,^20^). However, the stage at which mt-mRNAs are recruited to the mitoribosome, and the precise roles of the initiation factors in this process remain to be determined.

We have previously demonstrated that mitochondrial mtIF3 interacts with the mitochondrial SSU (28S, mtSSU) in a considerably different manner than its bacterial counterparts (20). Human mtIF3 forms an extensive network of contacts with the small subunit, which limits its flexibility and prevents binding of the initiator fMet-tRNA^fMet^. This suggests that mtIF3 must dissociate before fMet-tRNA^fMet^ and mRNA are accommodated to form the full initiation complex. Indeed, we could not detect mtIF3 and fMet-tRNA^fMet^ together on the mtSSU in our *in vitro* experiments (20), nor in the pre-initiation complexes isolated directly from the mammalian cells (21). Additionally, an *in vivo* murine model of mtIF3 deficiency has revealed that mtIF3 is indispensable for mitochondrial function, as its loss led to imbalanced protein synthesis and OXPHOS deficiency but does not completely shut down mitochondrial translation (22). Together, recent data show that mammalian mtIF3 function has highly diverged from its bacterial homologues.

Here, we further characterize mitochondrial initiation complex formation and to define the roles of mtIF2 and mtIF3 in this process, by employing novel biochemical *in vitro* assays together with cellular knockout models. Our studies reveal new insights into mitochondrial translation and highlight the importance of this process for mitochondrial homeostasis.

## MATERIALS AND METHODS

### Cell culture used for purification for *in vitro* translation initiation experiments

For *in vitro* translation initiation experiments, suspension-adapted and tetracycline-resistant human embryonic kidney cells lacking *N*-acetyl-glucosaminyltransferase I (HEK293S TetR GnTI−) were used. First, cells were grown adherently in T175 plates in DMEM + 10% FBS and 1× penicillin/streptomycin. Afterwards, 9 confluent plates were transferred to 500 ml FreeStyle 293 Expression Medium (Gibco, Life Technologies), supplemented with 5% fetal bovine serum (Gibco, Life Technologies) and 1× penicillin/streptomycin, and further cultured in 3 l Corning disposable spinner flask. The cells were passaged at a density of 3 × 10^6^ cells/ml to a density of 1.5 × 10^6^ cells/ml, by centrifugation at 1000 *g* for 10 min and complete exchange of the culture media. For mitoribosome preparation, a 6 l cell culture with a density of 6 × 10^6^ cells/ml was used.

### Generation of stable cell lines

To generate cell lines stably expressing the FLAG-tagged version of mtIF3 and mtIF2, an Flp-In T-Rex human embryonic kidney 293T (HEK293T, Invitrogen) cell line that permits doxycycline-inducible expression of the gene of interest in a dose-dependent manner was used. HEK293T cells were cultured at 37°C under 5% CO_2_ in DMEM (Dulbecco's modified Eagle's medium) supplemented with 10% (v/v) tetracycline-free fetal bovine serum (FBS), 1× penicillin/streptomycin (Gibco), 50 μg/ml uridine, 100 μg/ml zeocin (Invitrogen), and 10 μg/ml blasticidin S (Gibco). Parental HEK293T Flp-In T-Rex cells were seeded in a 10 cm dish, one day prior to transfection, to achieve 70–90% confluency. Co-transfection of the expression plasmid pcDNA5/FRT/TO-mtIF3-FLAG or mtIF2-FLAG and the Flp-recombinase plasmid, pOG44, was carried out using Lipofectamine 3000 (Invitrogen), according to the manufacturer's instructions. Selective antibiotics, 100 μg/ml of hygromycin (Invitrogen) and 10 μg/ml blasticidin S, were added 48 h post-transfection and culture media was replaced every 2–3 days.

### Generation of knock-out cell lines

The stable knock-out (KO) lines in HEK293T cells were established using the CRISPR/Cas9 system (23). Small guide RNA (sgRNA) pairs (Supplementary Table S1) were designed to generate an out-of-frame deletion and cloned into pSpCas9(BB)-2A-Puro (PX459) V2.0 vector. HEK293T cells grown in a six-well plate were transfected at 80% confluency using Lipofectamine 3000, as per the manufacturer's instructions. After 48 h of puromycin selection (1.5 μg/ml), cells were collected and serially-diluted single cells were plated onto multiple 96-well plates. The obtained clones were further screened by PCR and immunoblotting for verification of the targeted deletion and absence of the targeted protein, respectively.

To test viability of the WT, mtIF2 and mtIF3 knock-out cell lines in galactose, cells were seeded at a density of 50 000 cells/well of a six-well plate. The galactose media constituted DMEM without glucose (Invitrogen) supplemented with 0.9 g/l galactose, 2 mM GlutaMAX (Gibco), 1× sodium pyruvate, 10% (v/v) FBS and 1× penicillin/streptomycin. The cells were stained with trypan blue and counted at 48 h intervals up to 6 days with EVE™ Automated Cell Counter (NanoEnTek), according to the manufacturer's instructions.

### Preparation of mitoribosomal subunits and re-associated 55S for initiation studies

Human mitoribosomes were purified according to previously described protocols (24) with modifications. Briefly, HEK293 cells were pelleted by centrifugation at 1000 *g* for 10 min and washed with cold PBS. The pellet was then dissolved in Swelling Buffer (25 mM HEPES/KOH pH 7.5, 100 mM KCl, 20 mM Mg(OAc)_2_, 2 mM DTT) for 20 min at 4°C. Afterwards, the buffer was supplemented with sucrose and mannitol to a final concentration of 70 mM sucrose and 210 mM mannitol, and the cells were disrupted with 10 strokes in a 100 ml glass homogenizer. Nuclei and cell debris were pelleted by centrifugation at 1000 *g* for 10 min, and crude mitochondria were subsequently pelleted by centrifugation at 10 000 *g* for 10 min. Mitochondria were dissolved in Mitochondria Isolation Buffer (25 mM HEPES/KOH pH 7.5, 100 mM KCl, 20 mM MgOAc, 70 mM sucrose, 210 mM mannitol, 2 mM DTT, supplemented with cOmplete protease inhibitors) and treated with 10 U/ml Dnase I for 20 min on ice. After washing with Mitochondrial Isolation Buffer, 2 volumes of Lysis Buffer (25 mM HEPES–KOH pH 7.45, 100 mM KCl, 20 mM MgOAc, 2% Triton X-100, 2 mM DTT supplemented with cOmplete protease inhibitors and RNase inhibitors) were added and incubated on ice for 15 min at 4°C. The membranes were centrifuged at 30 000 *g* for 20 min, and the mitochondrial lysate was afterwards added on top of a 10–30% sucrose gradient in Ribosome Isolation Buffer (25 mM HEPES/KOH pH 7.5, 100 mM KCl, 20 mM MgOAc, 2 mM DTT) and centrifuged for 21 h at 21 000 rpm in an SW21 rotor. The gradients were then fractionated with a Biocomp Fractionator (Supplementary Figure S1D, I), 55S monosomes were pelleted and dissolved in Dissociation Buffer (20 mM HEPES/KOH pH 7.6, 300 mM KCl, 5 mM MgCl_2_, 1 mM DTT) for 5 h at 4°C, overlaid on top of a 10–30% sucrose gradient prepared in Dissociation Buffer, and centrifuged in an SW41 rotor for 21 h at 21 000 rpm. The gradient was subsequently fractionated using a Biocomp Piston Fractionator (Supplementary Figure S1D, II), the samples corresponding to the small and large ribosomal subunit were pooled individually and centrifuged in an SW60 rotor for 16 h at 55 000 rpm. To prepare re-associated ribosomes, the pellets corresponding to mitoribosomal subunits were dissolved in Re-association Buffer (20 mM HEPES–KOH pH 7.6, 30 mM KCl, 30 mM MgCl_2_, 1 mM DTT). The small and large ribosomal subunits were then mixed in a 1/1 ratio and incubated for 1 h at 37°C and 15 min on ice. The re-association reaction was overlaid on top of a 10–30% sucrose gradient prepared in the Re-association Buffer and centrifuged in an SW41-Ti rotor for 21 h at 21 000 rpm. The gradient was fractionated using a Biocomp Piston Fractionator (Supplementary Figure S1D, III), and the samples corresponding to the monosome were pooled and centrifuged in a TLA100.4 rotor for 16 h at 55 000 rpm.

### Modification of mitoribosomes

#### *In vitro* crosslinking

Crosslinking of mitoribosomes was achieved using published protocols (18), with minor modifications, in a buffer containing 50 mM HEPES/KOH, pH 7.6, 30 mM KCl, 10 mM MgCl_2,_ supplemented with 10 mM dimethyl suberimidate (DMS) (Crosslinking Buffer), pH 8.5. Re-associated ribosomes (1 μM) were incubated in Crosslinking Buffer for 2.5 h at 37°C, and the reaction was then stopped by the addition of 0.1 volume of 1 mM Tris–HCl, pH 7.6. To purify the fraction containing non-dissociable mitoribosomes, the sample was dialyzed against Dissociation Buffer overnight, overlaid on top of a 10–30% sucrose gradient in Dissociation Buffer, and centrifuged in an SW41 rotor for 21 h at 21 000 rpm. The gradient was fractionated, and the 55S fraction was pooled and centrifuged at 55 000 rpm in a TLA-55 rotor for 16 h. The pellet consisting of crosslinked ribosomes was dissolved in Initiation Buffer and used for further studies.

#### Fluorescent labeling of 55S mitoribosomes

Mitoribosomes (500 nM) were labeled at the surface accessible lysines, by incubation with Cy5-succinimidyl ester (2 μM) in Labeling Buffer (50 mM HEPES (pH 7.5), 100 mM KCl, 15 mM MgCl_2_) for 30 min at 37°C. To purify the labeled ribosomes, the reaction was overlaid on top of 1 ml sucrose cushion prepared in Labeling Buffer and centrifuged for 2 h at 55 000 rpm in a TLA-100.4 rotor. The pellet, consisting of labeled ribosomes, was dissolved in 20 μl Re-association Buffer and centrifuged at 14 000 rpm for 5 min in an Eppendorf centrifuge to remove the aggregates. The supernatant was then used fresh for further studies. Using this method, we were able to attach an average of 6 Cy5 dyes per monosome.

### Preparation of translational factors, *MTCO*2 mRNA, *E. Coli* fMet-tRNA^Met^_i_

Codon-optimized (Genscript) DNA constructs corresponding to the mature form of human mtIF2 (amino acids 38–727) and human mtIF3 (amino acids 32–278) were cloned into a pET-24b vector (Novagen). Both constructs were expressed in Rosetta 2 cells (EMD chemicals) at 25°C for 16 h in Magic Media (Thermo Fisher Scientific). After lysis, the proteins were purified over a His-Select Ni^2+^ resin (Sigma-Aldrich) and dialyzed against H-0.2 (25 mM Tris–HCl pH 7.4, 0.5 mM EDTA, 10% glycerol, 1 mM dithiothreitol, 200 mM NaCl). Further purification was conducted over a HiLoad 16/60 Superdex 200 pg gel filtration column (GE Healthcare) in buffer H-0.2 lacking glycerol.

C-terminal His-tagged *E. coli* EF-Tu was cloned into a pET15b plasmid and overexpressed in BL21(DE3) cells for 3 h at 37°C. The cells were resuspended in EF-Tu purification buffer A (50 mM Tris–HCl pH 7.6, 60 mM NH4 Cl, 7 mM MgCl_2_, 7 mM β-mercaptoethanol, 15 μM GDP, 15% glycerol) and lysed by sonication. After centrifugation at 20 000 rpm in an SS34 rotor, the supernatant was loaded onto a Co(II)-Sepharose resin (bed volume 3 ml), pre-equilibrated with EF-Tu purification buffer A, and incubated for 1 h at 4°C. The column was washed twice with 30 ml of EF-Tu purification buffer A,10 ml of EF-Tu purification buffer B (50 mM Tris–HCl pH 7.6, 60 mM NH4Cl, 7 mM MgCl_2_, 300 mM KCl, 7 mM β-mercaptoethanol, 15 μM GDP, 15% glycerol) and 10 ml of EF-Tu purification buffer C (50 mM Tris–HCl pH 7.6, 60 mM NH4Cl, 7 mM MgCl_2_, 300 mM KCl, 20 mM imidazole, 7 mM β-mercaptoethanol, 15 μM GDP, 15% glycerol). Subsequently, EF-Tu was eluted using 10 ml EF-Tu elution buffer (50 mM Tris–HCl pH 7.6, 60 mM NH4Cl, 7 mM MgCl_2_, 300 mM KCl, 300 mM imidazole, 7 mM β-mercaptoethanol, 15 μM GDP, 15% glycerol). The sample was dialyzed overnight against EF-Tu storage buffer (50 mM Tris–HCl pH 7.6, 100 mM NaCl, 10 mM MgCl_2_, 15μM GDP) and concentrated using Amicon Ultra ultrafiltration units (MWCO: 10,000).

The first 30 nucleotides of mitochondrially encoded cytochrome C oxidase 2 (*MTCO2*) mRNA with the sequence: 5′-AUGGCACAUGCAGCGCAAGUAGGUCUACAA -3′, labeled with Cy3 at the 3’ end, was purchased from Sigma-Aldrich.

The leaderless mRNA with the sequence 5’ AUG UUC AGA CAA GCG CAA GUA GGU CUA CAA 3’ used for dipeptide synthesis was ordered from Integrated DNA Technologies.

The unlabeled *Escherichia coli*, fMet-tRNA^fMet^ was generously provided by Prof. Marina Rodnina. *E. coli* (^35^S) fMet-tRNA^fMet^ was isolated from bulk *E. coli* MRE600 by hybridization to immobilised complementary oligoDNA. Yeast tRNA^Phe^ was purchased from Sigma. Charging with the cognate fMet and Phe amino acids, respectively, was performed as previously described (25).

### *In vitro* translation assays

All *in vitro* translation assays were carried out in assay buffer containing 50 mM Tris–HCl, pH 7.6, 30 mM KCl, 10 mM MgCl_2_, 5 mM GTP, 1 mM dithiothreitol DTT, 1mM spermidine, 0.1 mM spermine.

### mRNA binding assays

Reactions containing 200 nM 55S mitoribosomes or 28S subunits were supplemented with 240 nM mtIF3, 240 nM mtIF2, 240 nM *E. coli* fMet-tRNA^fMet^ and 400 nM *MTCO2* mRNA labeled with Cy3 at the 3’ end, incubated at 37°C for 30 min and on ice for 15 min. To purify the initiation complex, the reaction mixture was overlaid on top of a 10–30% sucrose gradient and centrifuged for 21 h at 21 000 rpm in an SW41 rotor. The optical density of the gradients was measured at 260 nm, and the presence of the Cy3-labeled mRNA was monitored by fluorescence spectroscopy using the same settings as described below (Fluorescence measurements and analysis). To qualitatively analyze the binding of the mRNA to ribosomal complexes resolved in the sucrose gradient, the absorbance at 260 nm and maximum fluorescence emission values of each fraction were normalized to the values detected for the first collected fraction. The fractions corresponding to the 55S ribosomes or 28S subunits peaks, identified by absorbance at 260 nm, were pooled and concentrated to 50 μl using a Vivaspin 500 centrifugal concentrator. Initiation complex formation was calculated based on molar concentrations of mitoribosomes (determined from absorption at 260 nm) and mRNA (as described in ‘Fluorescence measurements and analysis’).

For initiation reaction with crosslinked mitoribosomes, no mtIF3 was added as mtIF3 has been shown to possess partial dissociation activity (26).

### Filter binding assays

Ribosome complexes (50 μl) containing 200 nM 55S ribosomes or 28S subunits were assembled with (^3^H) *E. coli* fMet-tRNA^fMet^ in the same conditions as described above unless otherwise described. After incubation for 30 min at 37°C, the reactions were directly applied to nitrocellulose filters, which were then washed with 3 ml ice-cold assay buffer containing 20 mM Mg^2+^ to wash unbound (^3^H) fMet-tRNA^fMet^ tRNAs. The filters were dried for 15 min at 50°C and the radioactivity was measured in a liquid scintillation counter. A background corresponding to reactions in absence of mRNA was subtracted from each measured value.

### Co-sedimentation assays

To prepare dipeptide fMet-Phe, elongation reactions contained 0.2 μM 55S ribosomes, 1μM (^35^S) *E. coli* fMet-tRNA^Met^ and (^3^H) *Saccharomyces cerevisiae* Phe-tRNA^Phe^, 1 μM EF-Tu. After 1 h incubation at 37°C, the reaction was overlayed on top of a 1.1 M sucrose in assay buffer and centrifuged at 110 000 rpm for 2 h in an S140-AT rotor. The resulting pellet was washed with 1 ml cold assay buffer and dissolved in 20 μl assay buffer. The concentration of ribosomes was determined by measuring the absorption at 260 nm, and the ^35^S and ^3^H signal were measured in a liquid scintillator counter.

### *In vitro* transcription and biotinylation of the *MTCO2* mRNA

The *in vitro* transcribed *MTCO2* mRNA (prepared as in (15)) was biotinylated by enzymatically attaching biotinylated pCp at the 3′end using the T4 RNA ligase. To this end, 100 ng *MTCO2* mRNA was incubated with a 10 times excess of bio-pCp and 1 U/μl T4 RNA Ligase, in 1× Ligase Buffer, for 16 h at 16°C. Next, the bio*MTCO2* mRNA was cleaned with an RNeasy Mini Kit, and the concentration of the purified construct was measured spectroscopically with a Nanodrop.

### Purification of biotinylated initiation complexes

Samples containing 50 nM Cy5 55S monosomes or 50 nM Cy5 28S subunits were incubated with 75 nM bio*MTCO2* mRNA, in the same conditions as the *in vitro* initiation assays, with the exception that the incubation time was increased to 2 h. The initiation reactions were then incubated with 20 μl streptavidin-coated magnetic beads for 16 h at 18°C to allow binding of the biotinylated initiation complex to the beads. Next, the beads were washed three times with Initiation Buffer, and finally treated with 10 μg/ml RNase A for 1 h at 37°C to release the RNA bound to the magnetic beads. Cy5 fluorescence was afterwards measured in the Flow Through, Wash, and Eluate.

### Fluorescence measurements and analysis

Fluorescence spectra of the Cy3 fluorophore were measured with an FL-7000 fluorometer, at 25°C, in a 50 μl cuvette using the settings: excitation wavelength 620 nm, emission wavelength 625–800 nm, excitation and emission slits: 2.5 nm. The recorded spectra were corrected by subtracting the spectra of a reaction prepared in the same conditions, using unlabeled components. In order to convert the fluorescence intensity into concentration, a calibration curve was made using solutions of Cy3-labeled *MTCO2* mRNA of known concentrations, measured using the same settings (Supplementary Figure S1E).

### Confocal fluorescence detection

Confocal fluorescence detection was performed on a MicroTime 200 microscope (PicoQuant). Cy3 and Cy5 fluorophores were excited with two pulsed diode lasers of 510 nm (LDH-D-C-510) and 640 nm (LDH-D-C-640) with an average power of 20 μW and an UPLSAPO 60x/1.2NA objective (Olympus, Shinjuku, Japan). Fluorescence photons passed through a dual-band dichroic mirror (ZT514/640RPC, Chroma Technology, USA), 75 μm pinhole and were split into two detection pathways by a dichroic mirror (T620LPXR, Croma Technology). The red fluorescence emission passed through a bandpass emission filter (685/80, Chroma Technology) and was collected by a single-photon avalanche photodiode (τ-SPAD, PicoQuant). The green fluorescence emission passed through a bandpass filter (585/65, Chroma Technology) and was collected by a single-photon avalanche photodiode (COUNT T, Laser Components, USA).

First, we confirmed that the Cy3-*MTCO2* mRNA in the initiation reaction was bound to the mitoribosome using Fluorescence Correlation Spectroscopy (FCS) and compared the theoretical diffusion coefficients of the free Cy3, Cy3-*MTCO2* mRNA and Cy3-mitoribosome (Supplementary Figure S2C) with the measured diffusion coefficients (Supplementary Figure S2D).

Single-molecule brightness-gated-two-color coincidence detection (BTCCD) was measured under picomolar concentration, the detection probability of a single molecule was *N* ≤ 0.01 to avoid chance coincidence detection. The concentration prior to each dilution was checked with FCS. Sample aliquots were changed every 40 min to minimize dissociation contribution. Total acquisition time for the single-molecule data set was between 2 and 4 h. All measurements were made using PEGylated glass slides to rule out unspecific binding of molecules and maintain constant concentration during BTCCD measurements. More details on BTCCD method is provided in the Supplementary Note.

### SDS-PAGE and immunoblotting

Cell lysates were solubilized in lysis buffer (50 mM Tris–HCl, pH 7.4 with 150 mM NaCl, 1 mM EDTA, 1% Triton X-100) supplemented with 1× protease inhibitor cocktail (Roche) and 5 mM MgCl_2_. Protein concentrations were measured using Pierce™ BCA Protein Assay Kit (Thermo Fisher Scientific). Equal amounts of proteins from the total cell lysates and protein fractions were separated on a 4–12% SDS-PAGE and transferred to the PVDF membranes by wet transfer. The membranes were blocked for 1 h using 5% non-fat Milk (Semper) with PBS and incubated overnight at 4°C with a specific primary antibody, before detection the following day with secondary HRP-conjugates using ECL (BioRad) and films or digitally using ChemiDoc Imaging System (BioRad). The list of primary and secondary antibodies is summarized in Supplementary Table S2.

### *In vivo* mitochondrial translation assay

*In vivo* labeling of the mitochondrial translation products was performed by incubating the cells in growth media lacking methionine and cysteine supplemented with 44 μCi/ml of [^35^S]-methionine-cysteine EasyTag™ EXPRESS35S Protein Labeling Mix (Elmer-Perkin), 10% dialyzed FBS, 100× pyruvate (Gibco), 100× glutamine (Gibco) along with 100 μg/ml emetine dihydrochloride (Sigma Aldrich) to inhibit the cytosolic protein synthesis. Briefly, following 30 min incubation of cells with [^35^S]-methionine at 37°C, labeled cells were pelleted, washed with PBS and lysed. Equal amounts of proteins (40 μg) were separated on 12% SDS-PAGE, which were then dried (2 h at 65°C) for exposure to a Phosphor screen before scanning with a Typhoon FLA 7000 (GE Healthcare) for signal quantification.

### Mitochondrial extraction

Mitochondria from the WT HEK293T cells, mtIF2 and mtIF3 KO cell lines, and cells stably expressing mtIF3.FLAG were isolated as previously described (27). Briefly, cells were harvested by centrifugation at 400 *g* for 3 min and swollen in an ice-cold hypotonic buffer (0.6 M mannitol, 100 mM Tris–HCl pH 7.5, 10 mM EDTA, 0.05% BSA). Additional disruption of the cells was performed mechanically with glass homogenizer. The solution was centrifuged at 400 *g* for 10 min to remove cell debris and nuclei from the mitochondria. Supernatant was collected and centrifuged at 11 000 *g* for 10 min. At this point, crude mitochondrial pellets were frozen at –80°C or used directly for the analysis.

### Sucrose density gradient analysis

The sedimentation of mitoribosomes was analyzed using sucrose density gradients. The isolated mitochondria were solubilized with the lysis buffer (10 mM Tris–HCl, pH 7.4, 50 mM KCl, 20 mM MgCl_2_, 1% Triton X-100, 1× protease inhibitor cocktail (Roche), 40 U/μl RNase inhibitor (Agilent). 700 μg of protein was loaded onto a linear 10–30% gradient containing 10 mM Tris–HCl, 100 mM KCl and 20 mM MgCl_2_ supplemented with 1x protease inhibitor cocktail (Roche) and centrifuged at 79 000 *g* for 15 h at 4°C (Beckman Coulter; SW41 rotor). The gradients were collected from the top into 20 fractions of 450 μl/fraction, and fractions 1–18 were analyzed by immunoblotting, with fractions 1 and 2 pooled together.

### RNA purification and quantitative real time-PCR (qRT-PCR)

Total RNA was purified from the whole cells using TRIzol reagent as per manufacturer's instructions and quantified via NanoDrop ND-1000 UV-Vis Spectrophotometer (Thermo Fisher Scientific). Reverse transcription to initiate cDNA synthesis for qRT-PCR analysis was carried out using a High-Capacity cDNA Reverse Transcription Kit (Applied Biosystems). cDNA (5–10 ng) was mixed with 1× TaqMan Universal PCR Master Mix (Applied Biosystems) and universal TaqMan-based probes (Applied Biosystems, Supplementary Table S3). The qRT-PCR was performed on a QuantStudio 6 Flex Real-Time PCR System (Applied Biosystems). The recommendations of the manufacturer were followed when performing the reverse transcription and real time-PCR.

### BN-PAGE analysis and in-gel catalytic activity assay

Mitochondrial pellets were resuspended in aminocaproic buffer (1.5 M aminocaproic acid, 50 mM Bis–Tris, pH 7.0), quantified using Qubit (Invitrogen) and treated with 4% (w/v) digitonin. The lysate was incubated for 20 min on ice and centrifuged to remove insolubilized material. Equal amounts of protein (50 μg) were separated on pre-cast 3–12% Bis–Tris blue native gels (Invitrogen). In-gel catalytic activity assays were performed for mitochondrial respiratory complex I (2 mM Tris–HCl pH 7.4, 0.1 mg/ml NADH and 2.5 mg/ml iodonitrotetrazolium chloride), complex II (4.5 mM EDTA, 0.2 mM phenazine methosulfate, 84 mM succinic acid, 2.5 mg/ml iodonitrotetrazolium chloride), complex IV (50 mM phosphate buffer pH 7.4, 0.5 mg/ml 3,3’-diamidobenzidine tetrahydrochloride (DAB), 1 mg/ml cytochrome c, 0.2 M sucrose and 20 μg/ml catalase) and complex V (3.76 mg/ml glycine, 5 mM MgCl_2_, 1% Triton X-100, 0.5 mg/ml lead nitrate, 2 mM ATP, pH 8.4). Staining of the gels was carried out at room temperature.

### Mitochondrial bioenergetics with seahorse XFe96

The Seahorse XFe96 Analyzer (Seahorse Bioscience) was used to measure the oxygen consumption rate. Cells (3 × 10^5^ cells per well) were plated in the XF 96-well cell culture microplate coated with poly-d-lysine (Sigma) and grown for 24 h in a standard medium. One hour before the assay, the growth medium was replaced with 40 μl of Seahorse XF DMEM medium and the plate was left to stabilize in a 37°C non-CO_2_ incubator. The oxygen consumption rate (OCR) was measured for each well three times, every 3 min. OCR was normalized to protein concentration following the BCA assay conducted according to the manufacturer's instructions.

### Mitoribosomal profiling

The ribosome profiling experiment was performed as described previously (28), with minor modifications. During the separation of ribosome-protected fragments (RPFs) on 15% denaturing polyacrylamide gel, RNA species migrating between 10 and 50 nt were excised from the gel.

### Computational analysis of MitoRibo-seq data

Illumina adaptor sequences were trimmed from reads using the FASTX-Toolkit (http://hannonlab.cshl.edu/fastx_toolkit/). Trimmed reads shorter than 30 nt, or longer than 35 nt in length, were discarded. The remaining reads were mapped sequentially to nuclear-encoded ribosomal RNA (rRNA); mitochondrial rRNA (mt-rRNA); mitochondrial tRNA (mt-tRNA); mitochondrial mRNA (mt-mRNA); other nuclear non-coding RNA (ncRNA); and the nuclear genome. Read mapping was performed using bowtie version 1 (29), with parameters -v 2 –best (i.e. maximum two mismatches, report best match).

To facilitate the direct comparison of mitochondrial gene expression in WT and mtIF3 KO^1^ libraries, counts of mitochondrial ribosome-protected fragments (mt-RPFs) per gene in a given library were expressed as a percentage of total mtRPFs in that library. Only mtRPFs with 5′ ends mapping in the positive-sense orientation between the first nucleotide of the start codon and 30 nt 5′ of the stop codon were counted, and regions where ORFs overlap in the bicistronic ATP8/ATP6 and ND4L/ND4 transcripts were excluded from the expression calculations due to ambiguous assignment of RPFs to ORFs in these regions.

### Mass spectrometry analysis of mtIF3 FLAG immunoprecipitations

Identification and quantification of co-immunoprecipitated proteins was carried out as described previously (27). Peptides were separated on a 25 cm, 75 μm internal diameter PicoFrit analytical column (New Objective) packed with 1.9 μm ReproSil-Pur 120 C18-AQ media (Dr. Maisch,) using an EASY-nLC 1200 (Thermo Fisher Scientific). The column was maintained at 50°C. Buffer A and B were 0.1% formic acid in water and 0.1% formic acid in 80% acetonitrile. Peptides were separated on a segmented gradient from 6% to 31% buffer B for 45 min and from 31% to 50% buffer B for 5 min at 200 nl/min. Eluting peptides were analyzed on QExactive HF mass spectrometer (Thermo Fisher Scientific). Peptide precursor m/z measurements were carried out at 60 000 resolution in the 300–1800 *m*/*z* range. The 10 most intense precursors with charge state from 2 to 7 only were selected for HCD fragmentation using 25% normalized collision energy. The *m*/*z* values of the peptide fragments were measured at a resolution of 30 000 using a minimum AGC target of 2e5 and 80 ms maximum injection time. Upon fragmentation, precursors were put on a dynamic exclusion list for 45 s. The raw data were analyzed with MaxQuant version 1.6.1.0 (30) using the integrated Andromeda search engine (31). Peptide fragmentation spectra were searched against the canonical sequences of the human reference proteome (proteome ID UP000005640, downloaded September 2018 from UniProt). Methionine oxidation and protein N-terminal acetylation were set as variable modifications; cysteine carbamidomethylation was set as fixed modification. The digestion parameters were set to ‘specific’ and ‘Trypsin/P,’ The minimum number of peptides and razor peptides for protein identification was 1; the minimum number of unique peptides was 0. Protein identification was performed at a peptide spectrum matches and protein false discovery rate of 0.01. The ‘second peptide’ option was on. Successful identifications were transferred between the different raw files using the ‘Match between runs’ option. Label-free quantification (LFQ) (32) was performed using an LFQ minimum ratio count of two. LFQ intensities were filtered for at least two valid values in at least one group and imputed from a normal distribution with a width of 0.3 and down shift of 1.8. Protein quantification was performed using limma (33). Mitocarta (34) annotations were added using the primary gene name and the first of the gene name synonyms of the oldest Uniprot ID with the highest number of peptides.

### Statistical analyses

Statistical significance was analyzed using Microsoft Excel, and a two-tailed unpaired Student's *t*-test was performed to identify statistically significant differences between the groups. The significance threshold was set at *P* < 0.05; indicated as * for *P* < 0.05, ** for *P* < 0.01 and *** for *P* < 0.001. The experimental results are presented as mean ± standard deviation (SD).

## RESULTS

### Leaderless mRNAs bind 55S mitoribosomes more efficiently than 28S subunits

**Figure 1.**
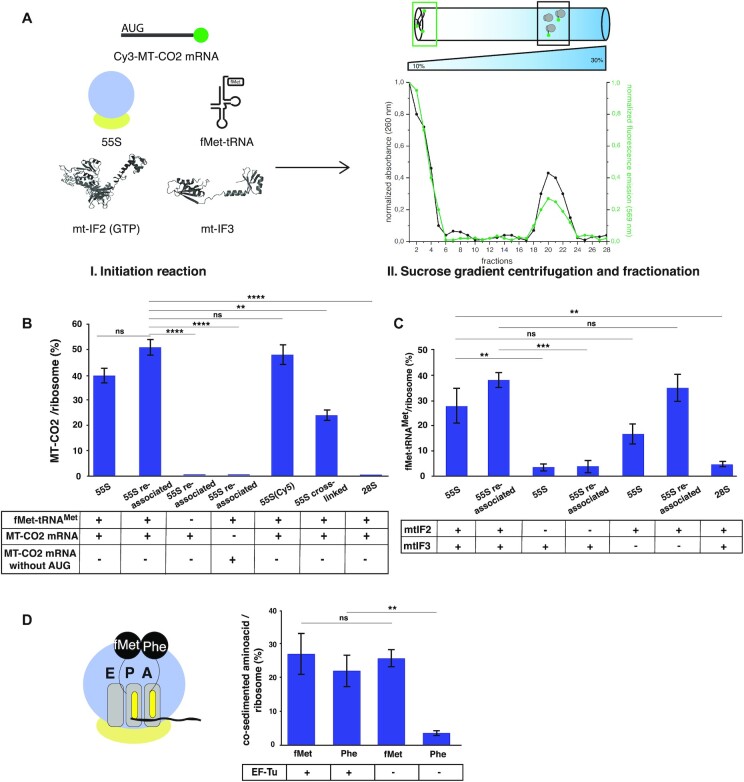
Detection of the mitochondrial translation initiation complex. (**A**) Binding of the Cy3 labeled *MT-CO2* mRNA to mitoribosomes. The optical density of the gradients was measured at 260 nm, and the presence of the Cy3-labeled mRNA was monitored by fluorescence spectroscopy and normalized to the signal in the first fraction. (**B**) Quantification of fMet-tRNA^fMet^-dependent binding of the Cy3-labeled *MTCO2* mRNA to human mitoribosomes or 28S subunit. Two negative controls: column 3: initiation reaction in the absence of fMet-tRNA^fMet^, and column 4: mRNA containing no start codon. Interestingly, crosslinked ribosomes were able to bind the Cy3-mRNA, suggesting that the dissociation into subunits is not a required step during mitochondrial translation initiation (column 6). No binding of the mRNA to the 28S was observed (column 7). (**C**) Quantification of *MTCO2* mRNA-dependent binding of the [^3^H]-fMet-tRNA^fMet^ to bovine liver mitoribosomes and 28S subunits and the effect of mtIF2 and mtIF3 in selection of the fMet-tRNA^fMet^ using a standard filter-binding assay. (**D**) Quantification of the dipeptide fMet-Phe synthesis by crude bovine liver mitoribosomes, measured by a co-sedimentation assay. In absence of EF-Tu, we observed a much reduced binding of Phe-tRNA^Phe^. The data represents mean values ± SD of three biological replicates.

We developed an *in vitro* reconstituted mitochondrial translation initiation system, using mammalian mitochondrial ribosomes, mitochondrial translation initiation factors mtIF2 and mtIF3, *E. coli* fMet-tRNA^fMet^ and a leaderless transcript (*MTCO2* encoding the first 30 ORF nucleotides), which contains the start codon AUG at the 5′ end. As a source of mammalian mitochondrial ribosomes, we used HEK293 cells and bovine liver tissue. We first analyzed the translation initiation efficiency of mammalian mitoribosomes by quantifying the binding of fluorescently labeled mRNA to human 55S mitoribosomes and 28S subunits. In addition, we quantified the initiation complex formation by measuring the binding of [^3^H] labeled initiator fMet-tRNA^fMet^ to bovine liver 55S mitoribosomes and 28S subunits. We observed similar translation initiation activity for both sources of mitoribosomes (Figure 1B and C).

We used two different procedures for the purification of 55S monosomes: crude ribosomes were prepared from mammalian mitochondria using a sucrose gradient (Supplementary Figure S1A), and re-associated ribosomes were isolated by dissociation of crude ribosomes and re-association of the purified small and large subunits (Supplementary Figure S1A, D). Crude ribosomes contain native co-purified mitochondrial tRNAs and mRNAs tRNAs, while re-association affords purification of empty mitoribosomes.

To observe binding of the mRNA to human mitoribosomes, we used an mRNA labeled with Cy3 at the 3′ end. After purification of the initiation complex through a sucrose gradient, the presence of ribosomes and mRNA was detected and the fraction of ribosomes carrying a Cy3-labeled mRNA was determined (Figure 1A). We observed that 40% of human crude 55S ribosomes carry a labeled mRNA (Figure 1B). In contrast, using *in vitro* re-associated 55S monosomes, led to a higher percentage of active mitoribosomes (*∼*50%) (Figure 1B), suggesting that natively-purified mitoribosomes may carry endogenous mRNA, tRNAs, and/or translational factors.

The presence of fMet-tRNA^fMet^ was essential for mRNA binding to 55S (Figure 1B). Moreover, we did not observe binding of an mRNA lacking the start codon to 55S ribosomes, proving that the AUG start codon at the 5′ is the unique binding site (Figure 1B). Next, we investigated whether fluorescent labeling of mitoribosomes had any impact on their binding affinity to *MTCO2* mRNA (labeled ribosomes were needed for the following experiments, as described below). After randomly labeling accessible lysines with NHS-functionalized Cy5 (Supplementary Figure S1B), mitoribosomes retained initiation activity (Figure 1B), indicating that the labeling conditions and presence of the Cy5 fluorophore did not inhibit recruitment of the mRNA. Importantly, we did not detect leaderless mRNA bound to the 28S subunit under the same conditions (Figure 1B).

To further investigate if leaderless mRNA can bind to 55S ribosomes, without their dissociation, we tested initiation activity of human mitoribosomes with covalently crosslinked subunits. Following crosslinking of accessible lysine residues (as described in (18)), most mitoribosomes remained as intact monosomes in dissociation buffer containing low Mg^2+^ and high K^+^ concentrations, while non-modified 55S mitoribosomes efficiently dissociated in these conditions (Supplementary Figure S1C). Following crosslinking, the sample containing 55S monosomes which did not dissociate in these conditions was pelleted and the *MTCO2* mRNA binding efficiency was determined, as described above. The crosslinked mitoribosomes retained initiation activity, although they were only half as active as re-associated mitoribosomes (Figure 1B, 25% versus 50%). This loss in activity is not unexpected, since the random crosslinker is likely to bind the ribosomal subunits in more than one location, with some of the introduced modifications potentially targeting regions essential for translation initiation.

We next performed additional filter-binding assays measuring mRNA-dependent [^3^H]-fMet-tRNA^fMet^ binding to mitoribosomes to form an initiation complex formed with 55S ribosomes and co-sedimentation experiments to determine whether the initiation complex is elongation-competent. In these experiments we used bovine liver as a source of mitochondria, which allows convenient purification of sufficient amounts of mitoribosomes. In addition, since it is currently challenging to purify and aminoacylate mammalian mt-tRNAs in the amounts required for *in vitro* studies, we used *E. coli* [^35^S]-fMet-tRNA^fMet^ and *S. cerevisiae* [^3^H]-Phe-tRNA^Phe^. Here it is important to note that although mt-Met-tRNA^Met^ and mt-Phe-tRNA^Phe^ do contain mt-specific modifications, they each maintain a classical secondary cloverleaf structure, including conserved tertiary interactions between the D-stem and variable loop (35).

We found a much higher binding efficiency of [^3^H]-fMet-tRNA^fMet^ to crude 55S ribosomes than to 28S subunits (Figure 1C), paralleling the mRNA binding results and in accord with the results obtained with the cross-linked 55S ribosomes. We also used the filter-binding assay to determine the *in vitro* role of mtIF2 and mtIF3 in selection of the [^3^H]-fMet-tRNA^fMet^ by both crude and re-associated 55S ribosomes. Omission of mtIF2 severely inhibited [^3^H]-fMet-tRNA^fMet^ binding to monosomes, showing that mtIF2 is crucial for the formation of the mitochondrial initiation complex. Omission of mtIF3 did not decrease initiation complex formation on reassociated ribosomes (Figure 1C). However, crude monosomes, which contain native mitochondrial tRNAs and mRNAs, showed a decrease in initiation activity in absence of mtIF3 (Figure 1C). This suggests that mtIF3 might facilitate the recycling of 55S crude ribosomal complexes by removing the native bound tRNAs and mRNAs before a subsequent initiation event.

Next, we determined whether the initiation complex formed with 55S ribosomes programmed with a 30-nucleotide leaderless mRNA (AUG UUC AGA CAA GCG CAA GUA GGU CUA CAA) and [^35^S]-fMet-tRNA^fMet^ is elongation-competent, by testing its ability to form ribosome-bound fMet-Phe-tRNA^Phe^ on mixing with [^3^H]-Phe-tRNA^Phe^ and recombinant *E. coli* elongation factor Tu (EF-Tu). Quantification of the [^35^S]-fMet and [^3^H]-Phe co-sedimenting with 55S ribosomes, the latter corrected by subtraction of a small background value determined in the absence of [^35^S]-fMet-tRNA^fMet^, showed that that 27% of 55S ribosomes contained [^35^S]-fMet-tRNA^fMet^ and 22% contained [^3^H]- Phe-tRNA^Phe^, indicating that ∼80% of the 55S initiation complex is ribosomes was active in EF-Tu-dependent dipeptide bond formation (Figure 1D).

To exclude the possibility that the sucrose gradient centrifugation or the limited size (30 nucleotides) of the mRNA accounted for our failure to observe formation of the initiation complex on the 28S subunits, we performed initiation reactions with Cy5-labeled human 55S monosomes or Cy5-labeled human 28S subunits with an *in vitro* transcribed full-length *MTCO2* mRNA. These experiments employed *MTCO2* mRNA biotinylated at the 3′ end (bio*MTCO2*), allowing initiation complexes to be purified using streptavidin-coated magnetic beads. A negative control with a bio*MTCO2* mRNA lacking the start codon was used to determine the non-specific binding of the transcript to ribosomes. The fluorescence intensity measured in the flow-through, wash and eluate, normalized to the total fluorescence intensity of each sample, revealed that Cy5-55S monosomes were recruited on the bio*MTCO2* mRNA, while the negative control showed no binding of Cy5-55S monosomes (Supplementary Figure S1F). In contrast, the Cy5-labeled 28S did not bind to any of the bio*MTCO2* mRNA constructs, indicating that, in our assay conditions, the mtSSU has a much lower affinity for the full *MTCO2* mRNA than 55S monosomes. Together, our *in vitro* experiments demonstrate that mitochondrial leaderless mRNAs bind 55S monosomes more efficiently than the 28S subunits. Moreover, initiator fMet-tRNA^fMet^ is essential for loading mRNA onto the full monosome to form an elongation-competent initiation complex.

### *In vitro*, mitochondrial translation can be initiated without exchange of ribosomal subunits

**Figure 2.**
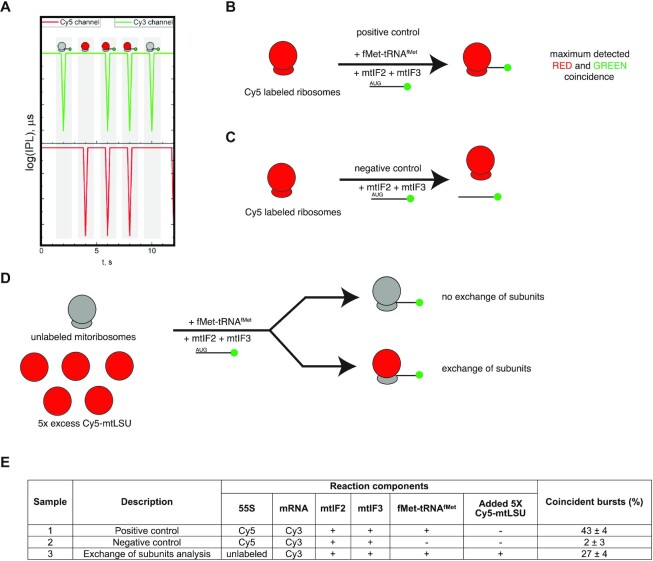
*In vitro*, mitochondrial translation can be initiated without exchange of ribosomal subunits. (**A**) Schematic concept of BTCCD. Non-associated, green-labeled molecules (Cy3-labeled *MTCO2* mRNA) and red-labeled molecules (Cy5-labeled ribosomes) give rise to non-coincident events, while double-labeled molecules (Cy5 mitoribosome bound to a Cy3 *MTCO2* mRNA) are identified as coincident events. (**B**) A sample containing initiation complex formed with Cy5-labeled human re-associated 55S monosomes (200 nM) and Cy3-labeled *MTCO2* mRNA (1 μM) was used as a positive control. (**C**) A negative control was prepared by incubating human re-associated Cy5-labeled 55S ribosomes (200 nM) with Cy3-labeled *MTCO2* mRNA (1 μM), in the absence of fMet-tRNA^fMet^. (**D**) A strategy for detecting the exchange of subunits during translation initiation. Unlabeled human re-associated 55S ribosomes (200 nM) were incubated with a 5-fold excess of Cy5-mtLSU (1 μM) and Cy3-labeled *MTCO2* (1 μM). The fraction of coincident Cy3 and Cy5 bursts was measured with BTCCD. (**E**) Fraction of coincident bursts detected with BTCCD for the samples described in (B)–(D).

Recently, a Brightness-Gated Two Color Coincidence Detection (BTCCD) method was developed to detect and quantify the fraction of associated biomolecules in solution, using standard confocal detection of fluorophore-labeled ribosomal subunits and mRNAs (Figure 2A, Supplementary Note 1) (36). This method was applied to quantify the fraction of bacterial ribosomes that dissociate during translation initiation *in vitro* (36), showing that most of the initiation events in bacteria occurred via the canonical dissociative mechanism (*30S binding initiation*). Here, we used a similar approach to investigate the frequency of exchange of mitoribosomal subunits during translation initiation in the mitochondrial *in vitro* system. For this purpose, we used BTCCD to determine the fraction of unlabeled human re-associated mitoribosomes, which exchanged the unlabeled large subunit during translation initiation of the Cy3-labeled *MTCO2* mRNA in presence of a 5-fold excess of Cy5-labeled mtLSU, relative to the 55S monosomes.

To estimate the maximum detected Cy3 and Cy5 coincidence in our system, we used a positive control consisting of Cy5-labeled 55S ribosomes that initiated Cy3-labeled *MTCO2* mRNA (Figure 2B). The 55S initiation complex was purified through a sucrose gradient to remove the excess of unbound Cy3-*MTCO2* mRNA (Supplementary Figure S2 A), and the fraction of coincident Cy5 and Cy3 bursts was determined to be 43 ± 4% (Figure 2E, Supplementary Figure S2E). This positive control accounted for incomplete labeling of the *MTCO2* mRNA, bleaching, and reversible dissociation of the mRNA from the mitoribosome due to dilution of the sample to picomolar concentration, possibly below *K*_d_ during single-molecule measurements.

A negative control in which Cy5-labeled 55S mitoribosomes were incubated with Cy3-labeled *MTCO2* mRNA in absence of fMet-tRNA^fMet^ (Figure 2C) showed a very much reduced fraction (2 ± 3%) of coincident bursts (Figure 2E, Supplementary Figure S2E), confirming that the initiator fMet-tRNA^fMet^ is essential for binding of the leaderless mRNAs to mitoribosomes.

Next, we determined the fraction of mitoribosomes which exchanged the large subunit during translation initiation. To be able to accurately quantify this fraction, we used a sample containing a 5-fold excess of Cy5-labeled mtLSU over unlabeled 55S ribosomes and Cy3-labeled *MTCO2* mRNA (Figure 2D). Excess Cy5-labeled mtLSU was added immediately before starting the reaction by incubation at 37°C in the same conditions as described above to allow translation initiation. After purification of the initiation complex through a sucrose gradient (Supplementary Figure S2B), we used BTCCD to determine the fraction of Cy3 and Cy5 coincident bursts, which consisted of initiation complexes containing a Cy5 labeled mtLSU and Cy3 labeled *MTCO2* mRNA. A complete rapid exchange of the unlabeled mtLSU with a labeled mtLSU would be predicted to give a coincident burst of (43 ± 4) × (5/6), or 36 ± 4%, but the observed value was 27 ± 4% (Figure 2E, Supplementary Figure S2E). Thus, under our conditions, rapid exchange of mtLSUs occurs prior to loading of Cy3-labeled *MTCO2* mRNA on the full mitoribosome for the majority (∼75%) of translation initiation events. However, 25% of events occurred on 55S monosomes without exchange of the mtLSU, further corroborating that translation initiation can occur on the monosome.

### mtIF2 is essential for initiation of mitochondrial translation

IF2 is a key player in the canonical mechanism of prokaryotic translation initiation, contributing to the fidelity of fMet-tRNA^fMet^ selection and catalyzing the joining of the 50S subunit to the 30S pre-initiation complex (PIC). The role of mammalian mitochondrial IF2 has only been investigated *in vitro* or in heterologous bacterial systems (14,37), and the consequences of its depletion on mitochondrial translation are unknown. To investigate the function of mtIF2, we constructed mtIF2 knockout HEK293T cell lines (KO) using CRISPR/Cas9-mediated genome editing (Supplementary Figure S3A). Deletion of mtIF2 was confirmed in two clonal lines, KO^1^ and KO^2^, via western blotting and PCR (Supplementary Figure S3B). As expected, KO cells were unable to proliferate in media with galactose as the sole carbon source, which forces cells to rely on OXPHOS for ATP production, indicating a mitochondrial dysfunction (Supplementary Figure S3C).

**Figure 3.**
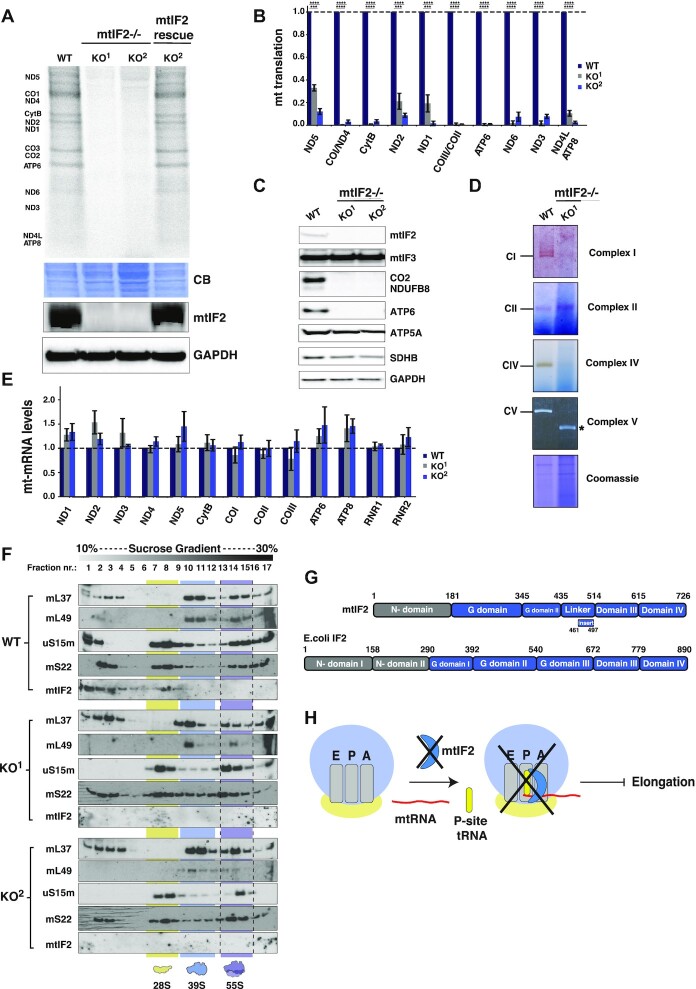
mtIF2 is essential for mitochondrial translation. (**A**) [^35^S]-methionine labeled nascent mitochondrial polypeptides from the WT, mtIF2 KO clones and KO^1^ cells re-expressing mtIF2.FLAG. Equal amounts (40 μg) of total cell lysates were separated by 12% SDS-PAGE and visualized by autoradiography. Coomassie Brilliant Blue (CBB) staining is provided to confirm equal protein loading and immunoblotting was used to show mtIF2 expression. GAPDH is used as a loading control. (**B**) Quantification of the [^35^S] labeled mitochondrial translation products from the WT and mtIF2 KO clones. Data represent the mean values ± SD from three independent biological experiments. (**C**) Steady-state levels of the OXPHOS subunits from WT and mtIF2 KO cell lines analyzed by the western blot. Proteins from the whole cell lysate were resolved by SDS-PAGE and transferred to a PVDF membrane, which was then probed with antibodies against mtIF3, mtIF2, mitochondrially-encoded (COXII, ATP6) and nuclear-encoded (NDUFB8, SDHB, ATP5a) subunits of the OXPHOS complexes. (**D**) In-gel activities of CI, CII, CIV and CV in mitochondrial protein extracts from the WT and mtIF2 KO clones. Coomassie blue stained gel is shown to indicate equal loading. This loss of ATP synthase integrity causes accumulation of the F_1_-containing assembly intermediate (*). (**E**) Quantitative real-time PCR analysis of mitochondrial mRNAs and rRNAs steady-state levels in mtIF2 KO cells relative to WT. Data is representative of the mean values ± SD from three independent experiments. (**F**) Mitoribosome sedimentation analysis on a sucrose density gradient (10–30%) for WT and mtIF2 KO cells. Mitochondria were isolated from cells, and lysates were loaded onto gradients. Following centrifugation, obtained fractions were analyzed by western blotting with antibodies against proteins of the mtLSU (uL3m and mL37), the mtSSU (uS15m, mS22) and mtIF2. Yellow, blue and purple color indicate sedimentation of the mtSSU, mtLSU and 55S, respectively. (**G**) Domain organization of human mtIF2 and *E.coli* IF2. N-terminal domains are shown in grey, insert represents mitochondria-specific insertion domain. (**H**) A model depicting the role of mtIF2 during translation initiation. mtIF2 is not essential for the monosome formation, however, in the absence of mtIF2, the complete initiation complex cannot be formed, as mtIF2 is likely needed for stabilization of the initiator tRNA and mRNA on the mitoribosome(15).

To determine if the growth phenotype of KO cells was directly attributable to a defect in mitochondrial translation, we performed metabolic labeling of mitochondrial translation products upon inhibition of cytosolic translation with emetine. The results of the [^35^S]-methionine/cysteine metabolic labeling indicated a near complete loss of mitochondrial translation in KO clones (Figure 3A and B), which was restored upon expression of mtIF2.FLAG cDNA (Figure 3A). The observed defect in mitochondrial translation was consistent with the steady-state levels of mitochondrially-synthesized OXPHOS components being greatly lowered in mtIF2 KO cell extracts, as analyzed by immunoblotting (Figure 3C). Additionally, an in-gel catalytic assay showed a striking deficiency in the enzymatic activity of the OXPHOS complexes I, IV and V. On the contrary, complex II which is comprised solely of nuclear-encoded subunits, displayed preserved activity consistent with wildtype levels (Figure 3D). The observed OXPHOS deficiency was also in agreement with markedly decreased oxygen consumption rates (Supplementary Figure S3D). Additionally, qPCR of mtIF2 KO cell lines revealed no significant changes to mt-mRNA or mt-rRNA steady-state levels (Figure 3E), implying that mtIF2 is exclusively involved in the regulation of translation, without affecting upstream events, including transcription.

A recent study suggested a role for mtIF2 in mtSSU assembly in Trypanosoma (37). To assess the importance of mammalian mtIF2 activity for human mitoribosome formation, we performed sucrose density gradients from mtIF2 KO cells. Interestingly, the sedimentation pattern of the mitoribosome in mtIF2-depleted cells revealed no global defect in the assembly of either individual subunits or of the full monosome (Figure 3F). Gradient centrifugations were performed either in standard conditions (20mM Mg(OAc)_2_) or with lower Mg(OAc)_2_ concentration (5mM, Supplementary Figure S3E) to prevent spontaneous subunit joining; however, we observed normally assembled monosomes in both conditions. Bacterial homologues of mtIF2 have long, N-terminal domains which have been shown to promote 70S initiation complex formation by stabilizing the sampling of the 50S subunit during its binding to the pre-initiation complex (13). In contrast, mammalian mtIF2 entirely lacks the domains corresponding to N terminal domains I and II of the *E. coli* IF2 (Figure 3G) (38). The lack of these domains may explain why mtIF2 appears not to be essential for the subunit joining.

Together, these data illustrate the necessity of mtIF2 for mitochondrial translation, most likely due to its involvement in the recognition of the initiator tRNA and stabilization of mRNA in the complete initiation complex, as previously suggested (15) (Figure 3H). In bacteria, efficient initiation with leaderless mRNAs can occur even in the absence of initiation factors (8). Our results demonstrate that this is not the case for mitochondrial translation.

### mtIF3 activity is required for translation of the ATP6 ORF

A number of functions have been assigned to bacterial IF3, including prevention of premature subunit association, and discrimination between the initiator and elongator tRNAs (39). It has also been suggested that IF3 is involved in the 70S scanning mechanism, by which the second cistron of bicistronic mRNAs is initiated (18). In mammalian mitochondria, mechanistic insight into the contribution of mtIF3 to initiation of translation is limited.

**Figure 4.**
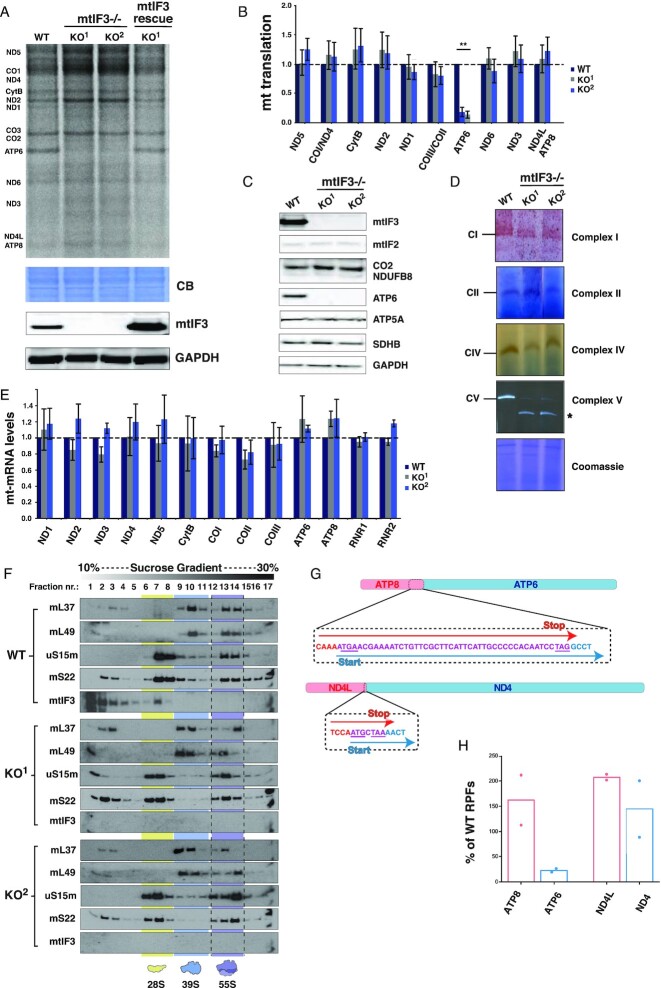
mtIF3 is essential for ATP6 synthesis. (**A**) [^35^S]-Met/Cys labeled nascent mitochondrial polypeptides from the WT mtIF3 KO cell lines and KO^1^ cells expressing mtIF3.FLAG or mtIF3-CTEΔ. Equal amounts of total proteins were separated on 12% SDS-PAGE, stained by Coomassie brilliant blue (CBB) to verify equal loading and visualized by autoradiography. mtIF3 expression is shown by the western blot with GAPDH serving as a loading control. (**B**) Quantification of the [^35^S]-Met/Cys labeled mitochondrial translation products from WT and mtIF3 KO clones. Data represent mean values ± SD from 5 independent biological replicates. (**C**) Steady-state levels of the OXPHOS subunits from WT and mtIF3 KO cell lines were analyzed by western blot. Proteins from the whole cell lysate were resolved by SDS-PAGE and transferred to the PVDF membranes, which were then probed with the antibodies against mtIF3, mtIF2, mitochondrially-encoded (COXII, ATP6) and nuclear-encoded subunits (NDUFB8, SDHB, ATP5a) of the OXPHOS complexes. GAPDH was used as a loading control. (**D**) BN-PAGE analysis and in-gel staining of CI, CII, CIV and CV activities in mitochondrial protein extracts from the WT and mtIF3 KO clones. Coomassie blue stained gel is shown as a loading control. *-indicates the F_1_-containing CV assembly intermediate. (**E**) Quantitative real-time PCR analysis of the mitochondrial mRNAs and rRNAs steady-state levels in mtIF3 KO cells relative to WT. Data is representative of the mean values ± SD from three independent experiments. (**F**) Mitoribosome sedimentation analyses on a sucrose density gradient (10–30%) in WT and mtIF3 KO cell lines. The yellow, blue and purple colors indicate the sedimentation of the mtSSU, mtLSU and 55S, respectively. (**G**) Schematics of the human mitochondrial bicistronic mRNA transcripts ATP8/ATP6 and ND4/ND4L. The overlapping sequence of the bicistrons is shown below in purple. (**H**) Mitoribosome profiling analysis. Percentages of mitoribosome-protected fragments (mt-RPF) per gene, relative to the WT control, shown for mitochondrial bicistronic transcripts (ATP8/ATP6 and ND4L/ND4) from mtIF3 KO^1^ cells. Results represent data from two MitoRiboSeq experiments.

In a similar manner as described for mtIF2, we generated several mtIF3 KO cell lines (Supplementary Figure S4A and B) and two clones (KO^1^ and KO^2^) were selected for further analysis. Both KO lines showed a growth defect in galactose media and markedly reduced oxygen consumption (Supplementary Figure S4C and D, respectively), supporting that this factor is also essential for mitochondrial function, as previously shown (40). Analysis of mitochondrial translation using a [^35^S]-methionine/cysteine incorporation assay showed a dramatic decrease in the production of ATP6, which constitutes the second ORF of the bicistronic ATP8/ATP6 mRNA (Figure 4A and B). Interestingly, synthesis of other mitochondria-encoded proteins was largely unaffected or slightly increased. This phenotype was reversed by the expression of full-length mtIF3.FLAG in KO^1^ cells. Western blotting further confirmed the loss of ATP6 steady-state levels in mtIF3 KO lines, while the levels of other OXPHOS components were not changed (Figure 4C). Analysis of in-gel OXPHOS activity confirmed that mtIF3 KO cells had dramatically reduced levels of complex V (F_1_F_0_‐ATPase) activity, whereas complexes I, II and IV were unchanged or only slightly decreased (Figure 4D). No significant differences in the steady-state levels of mt-mRNAs in KO cells, compared to controls, were detected (Figure 4E).

Together, these results demonstrate that mtIF3 is essential for translation of ATP6, while other mitochondrial proteins can be translated in the absence of mtIF3. Thus, the mitochondrial defect observed in mtIF3 KO cells is likely due to the loss of ATP6 expression, resulting in impaired complex V generation.

ATP6 is translated from the second ORF of the bicistronic ATP8/ATP6 mRNA, with two ORFs overlapping by 46 nucleotides (Figure 4G). The region encompassing the start codon of ATP6 and stop codon of ATP8 is highly unstructured, making it accessible to mitoribosomes (24), but the mechanism of the internal start codon recognition remains unexplored. Another mitochondrial bicistronic transcript, ND4L/ND4, has a much shorter overlapping region of only 7 nucleotides (Figure 4G), suggesting that the mechanism of the second ORF recognition may be different from ATP8/6. In support of this suggestion, we did not detect major changes in KO cells either in ND4 levels using a [^35^S]-Met/Cys translation assay (Figure 4A), or in complex I activity (Figure 4D), both results indicating that mtIF3 may not be essential for ND4 translation.

To gain more insight into the expression of second ORFs in mitochondrial bicistronic transcripts, we employed mitoribosome profiling (MitoRibo-Seq) (28). Since the steady-state levels of mitoribosomal proteins and monosome formation were not affected by the absence of mtIF3 (Figure 4F), we were able to obtain footprints of active 55S mitoribosomes for profiling analysis. In mtIF3 KO cells, we detected a pronounced reduction in the abundance of mitoribosome-protected fragments (mt-RPF) on the ATP6 ORF relative to WT, whilst the abundance of mitoribosomes was increased on the ATP8 ORF, suggesting that mtIF3 deletion can severely impact the formation of initiation complexes on the downstream ATP6 mRNA (Figure 4H). The occupancy of mitoribosomes on the other mitochondrial transcripts was variable upon mtIF3 knockout (COX2, COX3 and ND3 decreased, ND4, ND5 and ND6 increased, Supplementary Figure S4E), suggesting that mtIF3 may be involved, to some extent, in the binding of mitoribosomes to leaderless mRNAs, though without having a major effect on their translation (Figure 4A and B). However, no other transcript was affected as dramatically as ATP6 (Supplementary Figure S4E), confirming that ATP8/ATP6 bicistronic mRNA requires mtIF3 for translation of the second ORF. The second most affected transcript in this respect was ND3, suggesting that the expression of ND3 might depend on the action of mtIF3. This can also explain a slight decrease in the measured complex I activity in absence of mIF3 (Figure 4D).

## DISCUSSION

Until recently, our understanding of mitochondrial translation initiation has mostly relied upon observations in homologous bacterial systems. Considering the α-proteobacterial origin of mitochondria, similarities between these two translation systems are expected. However, cryoEM studies (3,5,15,20) clearly show that the mitochondrial translation machinery differs considerably from that of its bacterial counterpart and is also highly divergent between eukaryote phyla (41), while essential mechanistic insights are missing due to the lack of an efficient *in vitro* translation system. Importantly, a recently published *in vitro* assay enables monitoring of protein production by human mitoribosomes, representing a significant advance in studies of mitochondrial gene expression (42). However, in this assay translation is only detectable if the mRNA template contains a fragment of cI mRNA from bacteriophage lambda at the 5′ end; hence, it cannot be used to study the mechanism of translation initiation of mitochondrial leaderless transcripts. In addition, the very low activity of the mitoribosomes reported in (42) renders this *in vitro* system unsuitable for many mechanistic studies.

Here, we developed an efficient *in vitro* system that allows quantification of the initiation activity of mitoribosomes, employing fluorescence and radioactivity methods to monitor the binding of the mRNA and the initiator fMet-tRNA^fMet^, respectively. This assay was partially adapted from the previous work (24); however, we observed a considerable increase in initiation efficiency from the reported 4% (24) to ∼30% measured in our study. Moreover, we extended the analysis and detected binding of the second tRNA to the A site in the presence of elongation factor EF-Tu, showing that the purified initiation complexes are active in peptide bond formation. Using this system, we demonstrated that leaderless mRNAs bind preferentially to the fully assembled 55S monosomes rather than small subunits. In addition, we characterized the *in vitro* roles of mtIF2 and mtIF3 in mitochondrial translation initiation, showing that while mtIF2 is essential for translation initiation, mtIF3 activity is dispensable, suggesting similarity with the non-canonical translation initiation of leaderless mRNA observed in bacteria (18,43,44). However, we observed a decrease in initiation activity in the absence of mtIF3 when the reaction was performed with the crude mitoribosomes (Figure 1C). This partial loss in activity is consistent with the notion that mtIF3 plays a role in ribosome recycling by facilitating removal of tRNA and mRNA from the mitoribosome. Paralleling our *in vivo* results with the mtIF3 KO experiments, this observation suggests that ribosome recycling catalysed by mtIF3 is essential for re-initiation of the ATP6 ORF.

Why do leaderless mRNA preferentially bind to the monosome? Since no ribosomal recruitment signals other than the start codon have been identified on leaderless mRNAs, the interaction of the start codon with the anticodon of initiator tRNA bound to the ribosome is essential for complex stability. In bacteria, IF3 is loosely associated with the small subunit, allowing for large-scale movements, which ensure fidelity of selection of initiator tRNA and the cognate start codon, essential for the canonical initiation pathway (39). In contrast, our recent structural study shows that mtIF3 has limited conformational flexibility within the mtIF3-28S complex, due to an extensive network of contacts with the mtSSU (20), which inhibits mtIF3 movement and binding of the initiator tRNA. Our results suggest that leaderless mRNAs have no anchoring point stabilizing their binding to the mtSSU, at least in the presence of mtIF3. Interestingly, in our recent study, where we depleted mtLSU to stabilise initiation complex formation on the mSSU, we detected stalled complexes (mtSSU: fMet-tRNA^fMet^: mRNA: mtIF2) that contained COX1, but no other transcripts (21). COX1 is the only mitochondrial transcript with 3 nucleotides at the 5′ end of the transcript, preceding the start codon. These 5′ UTR residues were stacked with their bases against each other and against amino acids of uS7m, which likely stabilized the binding to the mtSSU. A configuration of one or two UTR residues would not interact with uS7m in this region. Therefore, it is likely that other transcripts are preferentially interacting with monosomes or can be stabilized on the mtSSU via transcript-specific factors that are yet to be discovered.

An interesting feature of the mitochondrial genome is the existence of two bicistronic transcripts with overlapping ORFs. Overlapping gene pairs are abundant in archaea and bacteria, with ‘termination-re-initiation’ translational coupling being widely used for their expression (45). In these cases, the same ribosome (or at least the same small subunit) that terminates at an upstream gene initiates translation at an overlapping downstream gene. The efficiency of re-initiation negatively correlates with the distance between the stop and restart codons, as re-initiation requires the scanning of mRNA by the 70S ribosome (or the 30S subunit) and the probability of its dissociation increases with the distance (45).

In mammalian mitochondria, the ND4 and ND4L ORFs overlap by only 7 nucleotides, likely allowing the mitoribosome to slide back from the ND4L stop codon to the ND4 start codon, without the need for mitoribosome splitting. For ATP8/ATP6 the overlap length is considerably larger, at 46 nucleotides. Our data indicate that mtIF3 function is critical for ATP6 start codon recognition. This is evidenced by the analysis of mtIF3 KO cells showing that: (i) ATP6 nascent peptide production is decreased, as measured by [^35^S] mitochondrial translation assay; (ii) steady-state levels of ATP6 proteins are compromised; (iii) activity of complex V is dramatically reduced; and (iv) association of mitoribosomes with the ATP6 ORF is markedly reduced in mtIF3 KO cells, as revealed by mitoribosome profiling data.

*In vitro*, mitochondrial IF3 has a strong affinity for the mtSSU and does not bind to the full monosome (46), as also confirmed by our mtIF3.FLAG immunoprecipitations (Supplementary Figure S5). Inhibition of ATP6 synthesis by knockout of mtIF3 suggests two possible mechanisms by which mtIF3 promotes translation of the ATP6 transcript. mtIF3 might play a role in ribosome recycling after translation termination, by inhibiting premature rebinding of the mtSSU to a mtLSU before the start codon of the ATP6 transcript has been correctly recognized. Alternatively, or in addition, mtIF3 might play an active role in the recognition of the ATP6 start codon. We were unable to reconstitute *in vitro* translation initiation on the ATP6 ORF using the conditions presented here (*data not shown*), which could be explained by the requirement of other factors involved in the process. In fact, it has already been hypothesized that LRPPRC, a mitochondrial mRNA binding factor and global mtRNA chaperone, may be required to expose the internal start codon of bicistronic transcripts to the mitoribosome for translation initiation (47).

A more complete understanding of the *in vivo* roles of mtIF3 would be helpful in this regard. Work by Rudler *et al.* revealed that mtIF3 is essential for survival in mice, as the heart- and skeletal muscle-specific loss of mtIF3 led to cardiomyopathy (22). The authors identified uncoordinated mitochondrial protein synthesis in mice lacking mtIF3, but they did not focus on the effect of KO on bicistronic transcripts (22). Importantly, in the course of our study, another group reported decreased ATP6 translation under mtIF3 depletion (48), strengthening our conclusions.

Our mitoribosome profiling analyses in mtIF3 KO cells showed that apart from ATP6, mitoribosome occupancy of other transcripts was, to some extent, also affected by mtIF3 loss (Supplementary Figure S4E), suggesting a subtle but general dysregulation of mRNA-mitoribosome interactions. However, this did not lead to a visible effect on nascent protein production (Figure 4A). Interestingly, we observed a considerable decrease in mitoribosomal footprints on the ND3 transcript, suggesting that this mRNA may also be dependent on mtIF3 for its translation. This is also indirectly supported by recent studies reporting an association of the ND3 transcript with the mtSSU (49), and its unusual behavior in comparison to other transcripts when the assembly of the mtLSU was compromised (50). Further studies are necessary to understand the details of translation initiation on the ND3 transcript.

**Figure 5.**
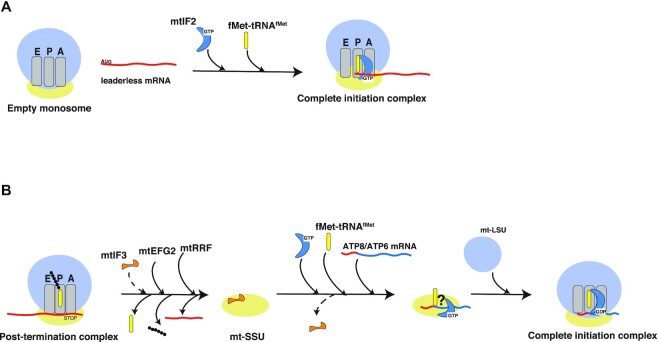
Model of translation initiation in mammalian mitochondria. (**A**) Leaderless mitochondrial transcripts are generally initiated by full 55S monosomes, in a process dependent on mtIF2, but independent on mtIF3. (**B**) The second ORF of the bicistronic ATP8/6 transcript is initiated by the mtSSU, in a process that requires mtIF3 function. mtIF3 may be essential either for stabilisation of the post-termination complex, allowing formation of the initiation complex on the mtSSU or it may play an active role in recognition of the internal start codon of the ATP6 mRNA.

Considering both our *in vitro* studies and the analyses of the cellular KO models, we propose a mechanism of translation initiation pathway in human mitochondria (Figure 5). Leaderless mRNAs are preferentially recruited to the 55S monosome and not the mtSSU, via a mechanism that does not require mtIF3 activity, and may depend on binding via the 5^’^end of mRNA (Figure 5A). In contrast, translation initiation of the ATP6 transcript requires mtIF3, which may play an essential role during translation recycling and/or translation initiation (Figure 5B).

Together, our data illustrate unique mechanisms of mitochondrial translation initiation and demonstrate the importance of this process for protein homeostasis and mitochondrial function. Overcoming the major limitations of present studies of mitochondrial gene expression—including finding efficient means of manipulation of the mitochondrial genome and the *in vitro* reconstitution of the complete mitochondrial translation system—will shed further light on mitochondrial translation initiation. A greater understanding of this area may aid the targeting of mitochondrial translation machinery by small molecule therapeutics, such as antibiotics and mitoribosome inhibitors (51,52).

## DATA AVAILABILITY

Mitoribo-seq data are deposited in the Gene Expression Omnibus (https://www.ncbi.nlm.nih.gov/geo/, accession number: GSE156937). Proteomic data are deposited in ProteomeXchange: PXD021015. The code for the BTCCD analysis is freely available at: https://doi.org/10.5281/zenodo.7414873.

## Supplementary Material

gkac1233_Supplemental_File
