# Supplementary material for: Translation initiation of leaderless and polycistronic transcripts in mammalian mitochondria
Source: Nucleic Acids Res. 2023 Jan 11;51(2):891–907. doi: 10.1093/nar/gkac1233 (PMC9881170; doi:10.1093/nar/gkac1233)
Supplement: gkac1233_Supplemental_File [file gkac1233_supplemental_file.pdf]

Supplementary Materials for

Translation initiation of leaderless and polycistronic transcripts in mammalian mitochondria

Cristina Remes[1,†], Anas Khawaja[2,3,†], Sarah F. Pearce[2,3], Adam M. Dinan[4], Shreekara Gopalakrishna[2,3], Miriam Cipullo[2,3], Vasileios Kyriakidis[2,3], Jingdian Zhang[2,3], Xaquin Castro Dopico[4], Olessya Yukhnovets[5,6], Ilian Atanassov[7], Andrew E. Firth[4], Barry Cooperman[1], Joanna Rorbach[2,3,*]

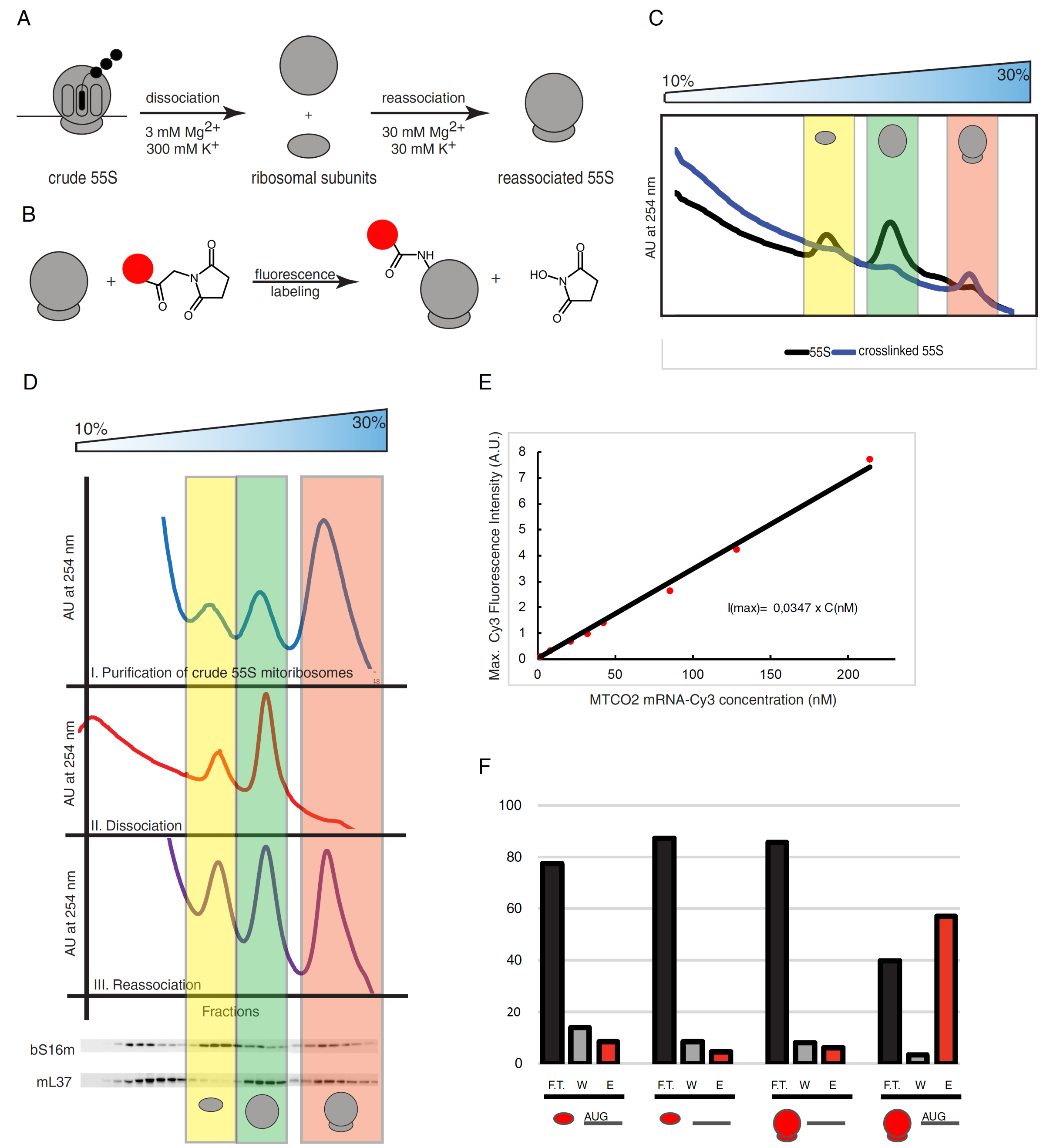

Supplementary Fig. 1. ***In vitro*** **translation initiation system.**

(A) Preparation of reassociated mitoribosomes for initiation studies. Crude 55S mitoribosomes purified from mitochondria were dissociated into subunits and the resulting subunits were re-associated to produce ‘empty’ mitoribosomes. (B) Fluorescent labeling of mitoribosomes. The accessible lysines were labeled with NHS-ester functionalized Cy5. (C) Dissociation of untreated mitoribosomes (black curve) versus covalently-crosslinked mitoribosomes (blue curve) following low [Mg^{2+}] and high [K^{+}] treatment. (D) Re-association of 55S ribosomes. Representative sucrose gradient fractionations of purified mitoribosomes (I), dissociation into subunits (II) and reassociation (III). Western blot analyses using antibodies against the mtSSU (bS16m) and mtLSU (mL37) were performed to support absorbance measurements (E)

Calibration curve to determine the concentration of the MTCO2 mRNA bound to the initiation complex. (F) Pulldown of Cy5-28S and Cy5-55S mitoribosomes assembled on the *in vitro* transcribed biotinylated bioMTCO2 mRNA immobilized on the streptavidin beads. The fluorescence of Cy5-28S or Cy5-55S was measured in the Flow Through (FT), Wash (W) and Eluate (E). A negative control was prepared in the same conditions, using mRNA without the start codon. (n=2)

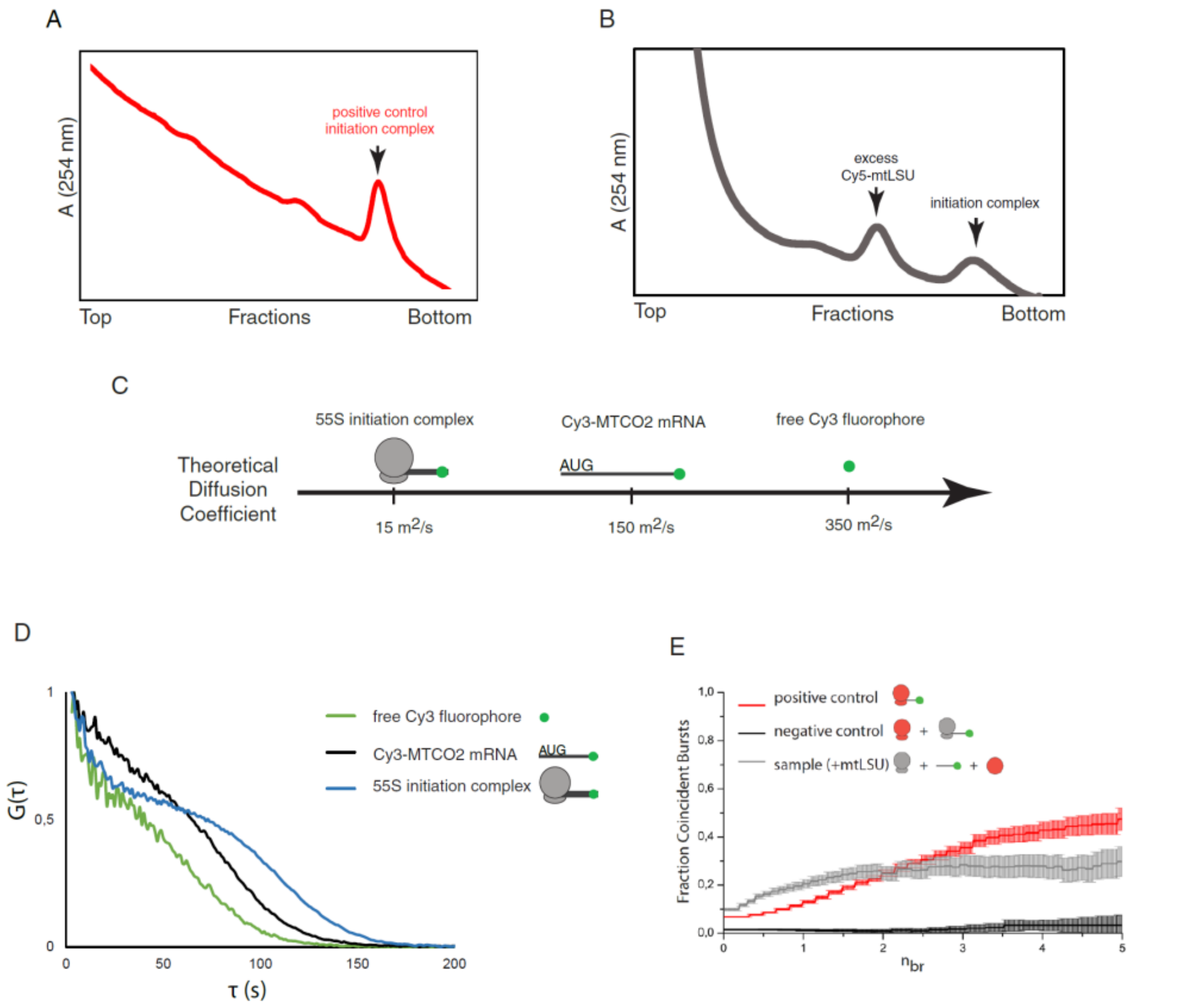

Supplementary Fig. 2. Investigation of mitoribosomal subunits exchange during translation initiation

(A) Sucrose gradient fractionation of the positive control for the BTCCD method (B) Sucrose gradient fractionation of the sample containing excess of Cy5 labeled mtLSU (C) Theoretical diffusion coefficients of the Cy3 labeled species (D) Fluorescence Correlation Spectroscopy analysis of the Cy3 labeled species proves that the Cy3 labeled MTCO2 mRNA is bound to the 55S ribosome in the initiation complex and does not dissociate in our measurement conditions. (E) BTCCD analysis for the positive control (red line), negative control (black line) and the sample (grey line).

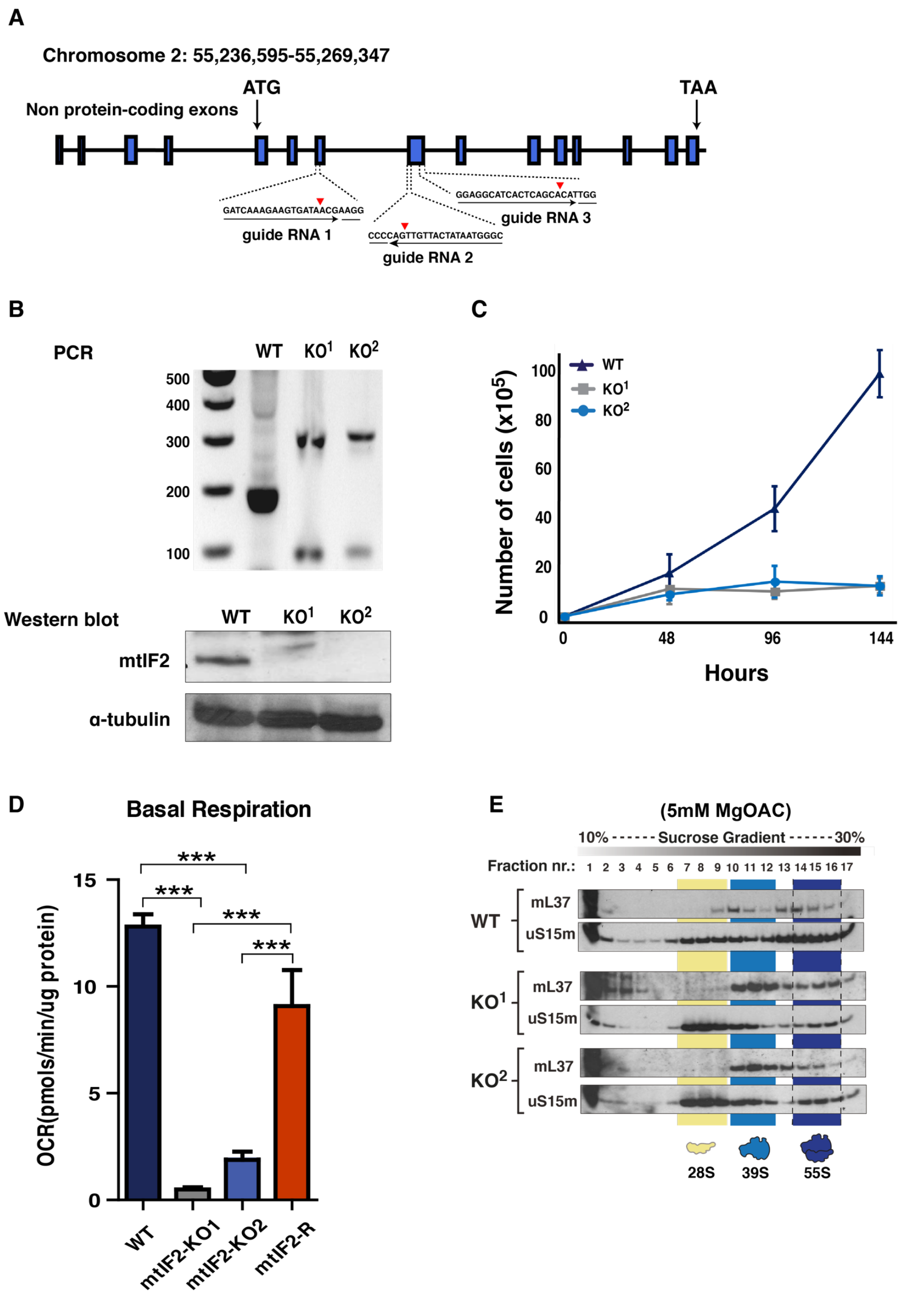

Supplementary Fig. 3. Characterisation of mtIF2 KO cells.

(A) Generation of the mtIF2 KO cells. Schematic representation of mtIF2 gene locus, indicating target sites of guide RNAs. Underlined sequences indicate the PAM (protospacer adjacent motif) sites and red arrows show the cleavage sites. (B) PCR to detect deletion of gRNA-targeted fragments. mtIF2 KO^1 is a result of transfection of HEK293T with gRNA set 1 and 3, and KO^2 is a result of the gRNAs 2 and 3. Further verification of the mtIF2 KO clones was performed by Western blot, with α-Tubulin used as loading control. (C) Growth curve of WT and mtIF2 KO clones cultured in DMEM containing galactose. Data show the mean values ±SD from three independent biological experiments. (D) Basal mitochondrial respiration (expressed in pmol of oxygen flux/mg of protein) for WT, mtIF2 KO, and rescue (mtIF2-R) cells measured

by Seahorse XFe96 Analyzer (n=3). (E) Mitoribosome sedimentation analysis on a sucrose density gradient (10-30%) for WT and mtIF2 KO cells performed in the presence of 5 mM $Mg(OAc)_2$. Mitochondria were isolated from cells, and lysates were loaded onto gradients. Following centrifugation, obtained fractions were analyzed by western blotting with antibodies against proteins of the mtLSU (mL37) and the mtSSU (uS15m) (n=3). Green, blue and purple color indicate sedimentation of the mtSSU, mtLSU and 55S, respectively.

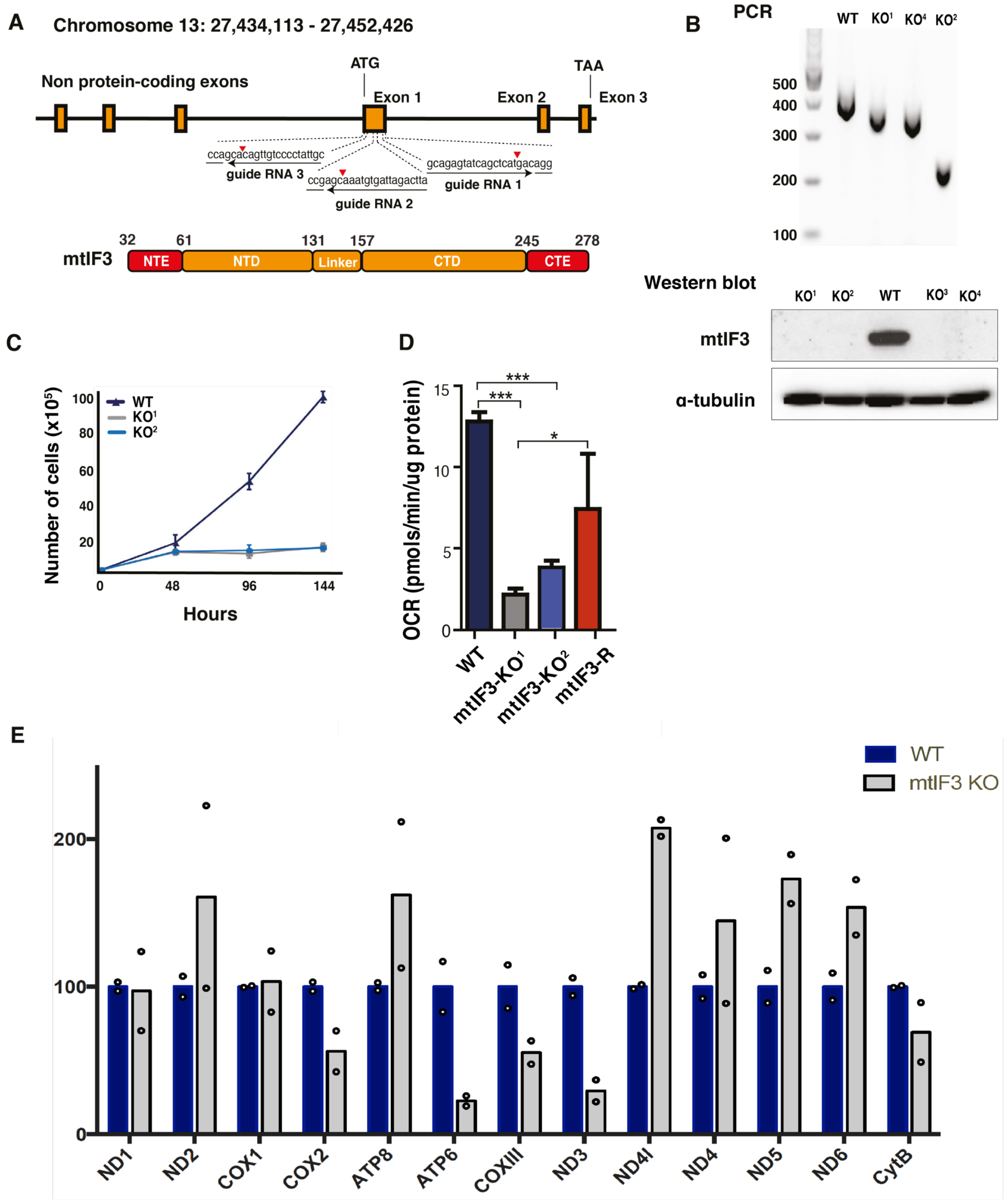

Supplementary Fig. 4. Characterisation of mtIF3 KO cells.

(A) Schematic representation of the location of the guide RNAs target sites on mtIF3 gene locus. Underlined sequences indicate the PAM site and red arrows show the site of cleavage. Bottom panel: Schematics of the mtIF3 domains (orange). The mitochondrial specific extensions on N- and C-terminus is shown in red. (B) The deletion of mtIF3 targeted fragments was confirmed by the PCR. mtIF3 KO^1 and KO^2 are a result of transfection of HEK293T cells with guide RNAs 1 and 2, and 1 and 3, respectively. Further verification of the mtIF3 KOs was performed by the Western blot. α-Tubulin was used as loading control. (C) Growth curves for WT and mtIF3 KO cells cultured in DMEM containing galactose. Data show the mean values

±SD from three independent biological experiments. (D) Basal mitochondrial respiration (expressed in pmol of oxygen flux / mg of protein) for WT, mtIF3 KO and rescue (mtIF3-R) cells measured by Seahorse XFe96 Analyzer (n=3). (E) Mitoribosome profiling of mtIF3 KO cells. Ribosome-protected fragments presented as a percentage of total mitochondrial RPF (mt-RPF) relative to the WT control (n=2).

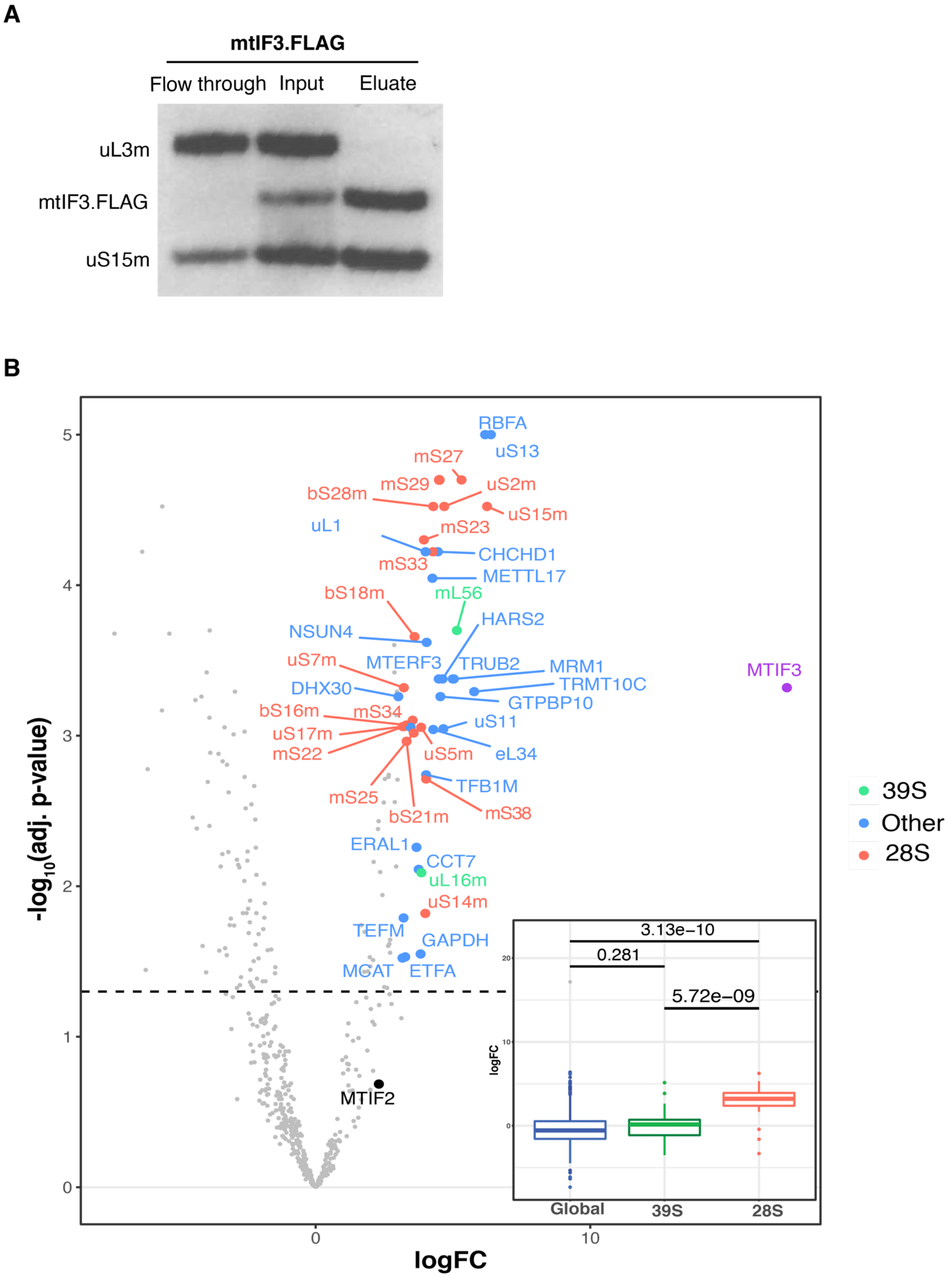

Supplementary Fig. 5. Interaction of mtIF3 with the 28S subunit.

(A) Western blotting analysis of mtIF3.FLAG pulldown. Input mitochondrial lysates and eluate of FLAG-IP from HEK293T expressing mtIF3.FLAG and control HEK293T without FLAG protein expression (WT) were resolved via SDS-PAGE, Western blotting was performed and subsequent membranes were probed with antibodies against FLAG and for proteins of either the mtLSU (uL3m) or mtSSU subunit (uS15m). (B) Mass spectrometry analysis of proteins interacting with mtIF3.FLAG. Following FLAG-IP, eluates were analyzed by label-free quantitative mass spectrometry (LFQ) (n=3). Volcano plot indicates proteins found in the Mitocarta 2.0 database. Inset: Boxplot displaying comparison of the logFC (log fold change) of proteins (as in main B) of the mtLSU or mtSSU in comparison to global proteins. Stated p-

values indicate pair-wise significance of difference in logFC between global proteins, mtLSU proteins or mtSSU proteins as determined via Welch’s unequal variances t-test .

SUPLEMENTARY MATERIALS AND METHODS

Preparation of mitoribosomal subunits and reassociated 55S for initiation studies

Human mitoribosomes were purified according to previously described protocols (Spremulli, 2007) with modifications.

Briefly, HEK293 cells were pelleted by centrifugation at 1000 *g* for 10 min and washed with cold PBS. The pellet was then dissolved in Swelling Buffer (25 mM HEPES/KOH pH 7.5, 100 mM KCl, 20 mM $Mg(OAc)_2$, 2 mM DTT) for 20 min at 4˚C. Afterwards, the buffer was supplemented with sucrose and mannitol to a final concentration of 70 mM sucrose and 210 mM mannitol, and the cells were disrupted with 10 strokes in a 100 mL glass homogenizer. Nuclei and cell debris were pelleted by centrifugation at 1000 *g* for 10 min, and crude mitochondria was subsequently pelleted by centrifugation at 10000 *g* for 10 min. Mitochondria was dissolved in Mitochondria Isolation Buffer (25 mM HEPES/KOH pH 7.5, 100 mM KCl, 20 mM MgOAc, 70 mM sucrose, 210 mM mannitol, 2 mM DTT, supplemented with cOmplete protease inhibitors) and treated with 10 U/mL Dnase I for 20 min on ice. After washing with Mitochondrial Isolation Buffer, 2 volumes of Lysis Buffer (25 mM Hepes-KOH pH 7.45, 100 mM KCl, 20 mM MgOAc, 2% Triton X-100, 2 mM DTT supplemented with cOmplete protease inhibitors and RNase inhibitors) were added and incubated on ice for 15 min at 4˚C. The membranes were centrifuged at 30000 *g* for 20 min, and the mitochondrial lysate was afterwards added on top a 10-30% sucrose gradient in Ribosome Isolation Buffer (25 mM HEPES/KOH pH 7.5, 100 mM KCl, 20 mM MgOAc, 2 mM DTT) and centrifuged for 21 h at 21000 rpm in a SW21 rotor. The gradients were then fractionated with a Biocomp Fractionator (Supplementary Fig. 1D, I), 55S monosomes were pelleted and dissolved in Dissociation Buffer (20 mM HEPES/KOH pH 7.6, 300 mM KCl, 5 mM $MgCl_2$, 1mM DTT) for 5 h at 4˚C, overlaid on top of a 10-30% sucrose gradient prepared in Dissociation Buffer, and centrifuged in a SW41 rotor for 21 h at 21000 rpm. The gradient was subsequently fractionated using a Biocomp Piston Fractionator (Supplementary Fig. 1D, II), the samples corresponding to the small and large ribosomal subunit were pooled individually and centrifuged in a SW60 rotor for 16 h at 55000 rpm. To prepare reassociated ribosomes, the pellets corresponding to mitoribosomal subunits were dissolved in Reassociation Buffer (20 mM HEPES-KOH pH 7.6, 30 mM KCl, 30 mM $MgCl_2$, 1mM DTT). The small and large ribosomal subunits were then mixed in a 1/1 ratio and incubated for 1h at 37˚C and 15 min on ice. The reassociation reaction was overlaid on top of a 10-30% sucrose gradient prepared in Reassociation Buffer and centrifuged in a SW41-Ti rotor for 21 h at 21000 rpm. The gradient was fractionated using a Biocomp Piston Fractionator

(Supplementary Fig. 1D, III), and the samples corresponding to the monosome were pooled and centrifuged in a TLA100.4 rotor for 16 h at 55000 rpm.

Mass spectrometry analysis of mtIF3 FLAG immunoprecipitations

Identification and quantification of co-immunoprecipitated proteins was carried out as described previously[41]. Peptides were separated on a 25 cm, 75 µm internal diameter PicoFrit analytical column (New Objective) packed with 1.9 µm ReproSil-Pur 120 C18-AQ media (Dr. Maisch,) using an EASY-nLC 1200 (Thermo Fisher Scientific). The column was maintained at 50°C. Buffer A and B were 0.1% formic acid in water and 0.1% formic acid in 80% acetonitrile. Peptides were separated on a segmented gradient from 6% to 31% buffer B for 45 min and from 31% to 50% buffer B for 5 min at 200 nl / min. Eluting peptides were analyzed on QExactive HF mass spectrometer (Thermo Fisher Scientific). Peptide precursor m/z measurements were carried out at 60000 resolution in the 300 to 1800 m/z range. The ten most intense precursors with charge state from 2 to 7 only were selected for HCD fragmentation using 25% normalized collision energy. The m/z values of the peptide fragments were measured at a resolution of 30000 using a minimum AGC target of 2e5 and 80 ms maximum injection time. Upon fragmentation, precursors were put on a dynamic exclusion list for 45 sec. The raw data were analyzed with MaxQuant version 1.6.1.0[43] using the integrated Andromeda search engine[44]. Peptide fragmentation spectra were searched against the canonical sequences of the human reference proteome (proteome ID UP000005640, downloaded September 2018 from UniProt). Methionine oxidation and protein N-terminal acetylation were set as variable modifications; cysteine carbamidomethylation was set as fixed modification. The digestion parameters were set to "specific" and "Trypsin/P," The minimum number of peptides and razor peptides for protein identification was 1; the minimum number of unique peptides was 0. Protein identification was performed at a peptide spectrum matches and protein false discovery rate of 0.01. The "second peptide" option was on. Successful identifications were transferred between the different raw files using the "Match between runs" option. Label-free quantification (LFQ)[45] was performed using an LFQ minimum ratio count of two. LFQ intensities were filtered for at least two valid values in at least one group and imputed from a normal distribution with a width of 0.3 and down shift of 1.8. Protein quantification was performed using limma[46]. Mitocarta[47] annotations were added using the primary gene name and the first of the gene name synonyms of the oldest Uniprot ID with the highest number of peptides.

Computational analysis of MitoRibo-Seq data

Illumina adaptor sequences were trimmed from reads using the FASTX-Toolkit (http://hannonlab.cshl.edu/fastx_toolkit/). Trimmed reads shorter than 30 nt, or longer than 35 nt in length, were discarded. The remaining reads were mapped sequentially to nuclear-encoded ribosomal RNA (rRNA); mitochondrial rRNA (mt-rRNA); mitochondrial tRNA (mt-tRNA); mitochondrial mRNA (mt-mRNA); other nuclear non-coding RNA (ncRNA); and the nuclear genome. Read mapping was performed using bowtie version 1 [42], with parameters -v 2 --best (i.e. maximum two mismatches, report best match).

To facilitate the direct comparison of mitochondrial gene expression in WT and mtIF3 KO[1] libraries, counts of mitochondrial ribosome-protected fragments (mt-RPFs) per gene in a given library were expressed as a percentage of total mtRPFs in that library. Only mtRPFs with 5′ ends mapping in the positive-sense orientation between the first nucleotide of the start codon and 30 nt 5′ of the stop codon were counted, and regions where ORFs overlap in the bicistronic ATP8/ATP6 and ND4L/ND4 transcripts were excluded from the expression calculations due to ambiguous assignment of RPFs to ORFs in these regions.

SUPPLEMENTARY NOTE (related to Supplementary Fig. 4)

Brightness-Gated Two-Color Coincidence Detection (BTCCD) is a further development of classical Two Color Coincidence Detection (TCCD) method[1]. It is used for quantification of fraction of associated and dissociated molecules in solution by means of dual-color single molecule confocal fluorescence detection. Detailed description of BTCCD analysis can be found in[2]. In brief, due to incomplete overlap of confocal volumes for different laser wavelength, molecule carrying both red and green fluorophores can be detected as single-labeled. In BTTCD analysis it is assumed that brighter bursts are collected from the molecules that spent longer time in the confocal volume or/and traveled through the center. Such bursts have higher probability to travel through both green and red confocal volumes. Coincidence fraction is calculated for varying range of brightness threshold nbr. Increasing nbr, calculated coincidence fraction will as well increase, until it saturates when only molecule trajectories that touched both volumes are left. Resulting coincidence value represents the real fraction of molecules that carry both red and greed fluorophores. Coincidence fraction for higher nbr corresponds to the increased overlap of two confocal volumes and, therefore, more accurate value. However, precision of the determined coincidence fraction decreases because the number of bursts entering analysis is lower for higher nbr. Optimal nbr, opt is defined as an intersection point between relative accuracy and relative precision. Most reliable coincidence fraction is defined as a coincidence fraction for nbr, opt.

[1]Orte, A., Clarke, R., Balasubramanian, S. & Klenerman, D. Determination of the fraction and stoichiometry of femtomolar levels of biomolecular complexes in an excess of monomer using single-molecule, two-color coincidence detection. Anal. Chem. (2006) doi:10.1021/ac061122y.

[2]Höfig, H. et al. Brightness-gated two-color coincidence detection unravels two distinct mechanisms in bacterial protein translation initiation. Commun. Biol. (2019) doi:10.1038/s42003-019-0709-7.

Table S1. List of guide RNAs

**sgRNA for mtIF2 exon 1**	Sequence (5➞3')
sgRNA_1_Top	CACCGGATCAAAGAAGTGATAACGA
sgRNA_1_bottom	AAACTCGTTATCACTTCTTTGATCC
Screen_deletion_exon1_mtIF2_forward	ATTATGTATATGAAGCTTTATTG
Screen_deletion_exon1_mtIF2_reverse	CTTCTTACAGCATCTTTATTTTTC
**sgRNA for mtIF2 exon 2**	
sgRNA_2_Top	CACCGGCCCATTATAGTAACAACTG
sgRNA_2_bottom	AAACCAGTTGTTACTATAATGGGCC
sgRNA_3_Top	CACCGGGAGGCATCACTCAGCACAT
sgRNA_3_bottom	AAACATGTGCTGAGTGATGCCTCCC
Screen_deletion_exon2_mtIF2_forward	GCCCCAGGCAGATCCAG
Screen_deletion_exon2_mtIF2_reverse	GTTAATTGTAGGCCCCAGGCAG
**sgRNA for mtIF3 exon 1**	
sgRNA_1_Top	CACCGGCAGAGTATCAGCTCATGAC
sgRNA_1_bottom	AAACGTCATGAGCTGATACTCTGCC
sgRNA_2_Top	CACCGTAAGTCTAATCACATTTGCT
sgRNA_2_bottom	AAACAGCAAATGTGATTAGACTTAC
sgRNA_3_Top	CACCGGCAATAGGGGACAACTGTGC
sgRNA_3_bottom	AAACGCACAGTTGTCCCCTATTGCC
Screen_deletion_mtIF3_forward	ATGGCTGCTTTTTTCTAAAGAGG
Screen_deletion_mtIF3_reverse	CAGTTTTGGGGTTCGCCTTCTCC

Table S2. List of Antibodies used for Western Blot

**Antibody**	**Company**	**Catalog no.**
Anti-FLAG	Abcam	ab1257
GAPDH	Abcam	ab8245
Alpha-tubulin	Sigma-Aldrich	T6199
VDAC/Porin	Abcam	ab14734
mtIF2	Abcam	ab197771
mtIF3	Proteintech Group	14219-1-AP

uS15m	Proteintech Group	17006-1-AP
uS17m	Proteintech Group	18881-1-AP
mS22	Proteintech Group	10984-1-AP
mS35	Proteintech Group	16457-1-AP
bS16m	Prestige, Sigma-Aldrich	HPA054538
uL3m	Prestige, Sigma-Aldrich	HPA043665
uL4m	Prestige, Sigma-Aldrich	HPA051261
uL15m	Proteintech Group	18339-1-AP
mL37	Prestige, Sigma-Aldrich	HPA025826
mL49	Proteintech Group	15542-1-AP
bL28m	Proteintech Group	21604-1-AP
SDHA	Abcam	ab14715
NDUFB8	Abcam	ab110242
MT-CO1	Abcam	ab14705
MT-CO2	Abcam	ab110258
ATP5a	Abcam	ab14748
ATP6	Proteintech Group	55313-1-AP
HRP secondary anti-rabbit	GE Healthcare	NA9340V
HRP secondary anti-mouse	GE Healthcare	NA9310V
HRP secondary anti-goat	Santa-Cruz	sc-2354

Table S3. List of TaqMan probes used for qRT-PCR

TaqMan probe	Assay ID
MT-ND1	Hs02596873
MT-ND2	Hs02596874
MT-ND3	Hs02596875
MT-ND4	Hs02596876
MT-ND5	Hs02596878
MT-ND6	Hs02596879
MT-CYTB	Hs02596867
MT-COX1	Hs02596864
MT-COX2	Hs02596865
MT-COX3	Hs02596866
MT-ATP6	Hs02596862
MT-ATP8	Hs02596863
MT-RNR1	Hs02596859
MT-RNR2	Hs02596860
